# Associations of erythrocyte polyunsaturated fatty acids with incidence of stroke and stroke types in adult Chinese: a prospective study of over 8000 individuals

**DOI:** 10.1007/s00394-022-02879-y

**Published:** 2022-04-21

**Authors:** Liang Sun, Huaidong Du, Geng Zong, Yu Guo, Yan Chen, Yiping Chen, Huiyong Yin, Pei Pei, Ling Yang, Qianqian Chu, Canqing Yu, Yixue Li, Jun Lv, He Zheng, Puchen Zhou, Junshi Chen, Liming Li, Zhengming Chen, Xu Lin

**Affiliations:** 1 Shanghai Institute of Nutrition and Health, University of Chinese Academy of Sciences, Chinese Academy of Sciences, 320 Yue-yang Rd, Shanghai 200031, China; 2 MRC Population Health Research Unit, Nuffield Department of Population Health, University of Oxford, Oxford, UK; 3 Clinical Trial Service Unit and Epidemiological Studies Unit, Nuffield Department of Population Health, Big Data Institute Building, University of Oxford, Old Road Campus, Oxford OX3 7LF, UK; 4 Chinese Academy of Medical Sciences, Beijing, China; 5 Department of Epidemiology and Biostatistics, School of Public Health, Peking University Health Science Center, Beijing, China; 6 Bio-Med Big Data Center, Key Laboratory of Computational Biology, CAS-MPG Partner Institute for Computational Biology, Shanghai Institute of Nutrition and Health, University of Chinese Academy of Sciences, Chinese Academy of Sciences, Shanghai, China; 7 Collaborative Innovation Center for Genetics and Development, Fudan University, Shanghai, China; 8 School of Life Sciences and Biotechnology, Shanghai Jiao Tong University, Shanghai, China; 9 China National Centre for Food Safety Risk Assessment, Beijing, China

**Keywords:** Polyunsaturated fatty acids, Ischemic stroke, Intracerebral hemorrhage, Prospective, Chinese

## Abstract

**Purpose:**

There is limited and inconsistent evidence about the relationships of erythrocyte polyunsaturated fatty acids (PUFAs) with stroke and stroke types, particularly in China where the stroke rates are high. We aimed to investigate the associations of different erythrocyte PUFAs with incidence of total stroke, ischemic stroke (IS), and intracerebral hemorrhage (ICH) in Chinese adults.

**Methods:**

In the prospective China Kadoorie Biobank, erythrocyte PUFAs were measured using gas chromatography in 10,563 participants who attended 2013–14 resurvey. After a mean follow-up of 3.8 years, 412 incident stroke cases (342 IS, 53 ICH) were recorded among 8,159 participants without prior vascular diseases or diabetes. Cox regression yielded adjusted hazard ratios (HRs) for stroke associated with 13 PUFAs.

**Results:**

Overall, the mean body mass index was 24.0 (3.4) kg/m^2^ and the mean age was 58.1 (9.9) years. In multivariable analyses, 18:2n–6 was positively associated with ICH (HR = 2.33 [95% CIs 1.41, 3.82] for top versus bottom quintile, *P*_trend_ = 0.007), but inversely associated with IS (0.69 [0.53,0.90], *P*_trend_ = 0.027), while 20:3n–6 was positively associated with risk of IS (1.64 [1.32,2.04], *P*_trend_ < 0.001), but not with ICH. Inverted-U shape curve associations were observed of 20:5n–3 with IS (*P*_nonlinear_ = 0.002) and total stroke (*P*_nonlinear_ = 0.008), with a threshold at 0.70%. After further adjustment for conventional CVD risk factors and dietary factors, these associations remained similar.

**Conclusion:**

Among relatively lean Chinese adults, erythrocyte PUFAs 18:2n–6, 20:3n–6 and 20:5n–3 showed different associations with risks of IS and ICH. These results would improve the understanding of stroke etiology.

## Introduction

Stroke is the second leading cause of death and permanent disability worldwide, with particularly high disease burden in many low- and middle-income countries [1, 2]. China has the world's highest age-standardized incident rate of stroke, with ~ 34 million prevalent cases and ~ 2.4 million new cases annually [1, 3, 4]. Compared to Western populations, a higher proportion of incident stroke in China was attributed to intracerebral hemorrhage (ICH) for reasons that are still poorly understood [1]. Dietary factors (e.g., low consumption of fruits, vegetables and coarse grains and high sodium intake) have been shown to play important roles in etiology of stroke, accounting for an estimated 65% of stroke-related disability-adjusted life years in China [3].

Previous prospective studies and clinical trials from mostly Western populations showed that substituting dietary polyunsaturated fatty acids (PUFAs) for saturated fatty acids (SFAs) was associated with reduced levels of total cholesterol and low-density lipoprotein cholesterol (LDL-C), and lower risk of stroke [5, 6]. However, the n-6 and n-3 classes of PUFAs appear to have opposing biological properties and can compete with each other for enzymes in endogenous conversion (Supplementary Fig. 1) and esterifying sites in membrane phospholipids [7–9]. Much higher consumption of n-6 PUFAs than n-3 PUFAs globally raised concerns about the potential long-term health consequences [10]. Compared with questionnaire-based assessment of PUFA intakes, objectively measured circulating PUFAs are not influenced by recall bias and, therefore, can reflect body PUFA levels more accurately, particularly for those essential PUFAs like 18:2n–6 (linoleic) and 18:3n–3 (α-linolenic), which are obtained exclusively from dietary intake rather from de novo synthesis. Unlike circulating PUFAs detected in other lipid fractions, erythrocyte PUFAs could reflect a relatively long-term average level and were highly correlated with PUFA composition in various tissues [11, 12]. However, only a few prospective studies have examined the associations of erythrocyte PUFAs with stroke, showing inverse associations of marine n-3 PUFAs with risk of ischemic stroke (IS) [13, 14], but null associations for n–6 PUFAs [15, 16]. In addition, these studies were conducted in Western populations and focused primarily on IS, with little data from China and other East Asian populations, where the stroke rates, proportion of different stroke types, dietary patterns [17] and genetic ability to metabolize certain PUFAs differed considerably from European-ancestry populations [18].

To fill the evidence gap, we present relevant data from the prospective China Kadoorie Biobank (CKB). The main objectives of the study were to (1) assess whether individual n-3 and n-6 PUFAs were independently associated with total stroke and stroke types (IS and ICH) and (2) to evaluate whether these associations were modified by conventional cardiovascular disease (CVD) risk factors (e.g. adiposity, blood pressure and blood lipids) and dietary factors.

## Methods

### Study population

The present study was based on participants who attended the CKB 2013–14 resurvey. Details of the CKB design and methods were described previously [19, 20]. In brief, the baseline survey of CKB took place between June 2004 and July 2008, all permanent residents aged 35–74 years from ten geographical diverse areas (five urban and five rural) were invited to participate during the period of time. These areas were deliberately selected in order to cover a wide range of risk exposures and disease patterns within China. About one in three of these invited adults participated in our baseline survey, which enrolled > 512,000 men and women. From August 2013 to September 2014, a representative subpopulation of ~ 5% survivors (~ 33,000) were invited to participate in the scheduled 2nd resurvey and ~ 76% (*n* = 25,239) attended.

### Data collection

At the 2013–14 resurvey, detailed information on sociodemographic status, lifestyle factors (smoking, alcohol drinking, and physical activity), dietary intake of major food groups (refined grain, coarse grain, red meat, poultry, fish, eggs, fresh vegetables, soya, fresh fruits and milk), and medical history were collected by interviewer-administered laptop-based questionnaires [21]. A range of physical measurements were undertaken, including standing and sitting height, weight, blood pressure, and heart rate. Non-fasting blood samples (and self-reported time since last meal) were collected for on-site tests of random blood glucose (Johnson & Johnson, New Brunswick, NJ, USA) and lipids (Mission Cholesterol Monitoring System, Acon Laboratories Inc, San Diego, CA), and long-term storages. The daily amount of physical activity (metabolic equivalent tasks [MET]-hr/day) was obtained by summing the MET-hours for activities related to occupation, commuting, housework, and non-sedentary leisure-time activities [22].

Ethical approvals for baseline survey were obtained from the Oxford Tropical Research Ethics Committee (OXTREC) at the University of Oxford and the Chinese Center for Disease Control and Prevention (CDC) Ethical Review Committee. Ethical approvals for the 2013–14 resurvey were obtained from OXTREC and the Chinese Academy of Medical Sciences/Peking Union Medical College Ethical Review Committee. Approval for baseline survey and the 2013–14 resurvey was also granted by the institutional boards at the CDCs in ten study areas. All study participants provided written informed consent.

### Measurement of erythrocyte fatty acids

The blood samples were collected into a 10-mL EDTA tube and stored at refrigerator for a few hours before transferring in cool boxes to local study laboratories for centrifuge and sub-aliquoting (into plasma, buffy coat and red cell). The red cell samples were stored at –80 °C until analysis during 2016–2018. Of the samples collected, a total of 10,933 (~ 1100 per area) were randomly selected to have erythrocyte fatty acids measured.

Erythrocyte fatty acids were measured by gas chromatography with flame ionization detector as previously described [23]. Briefly, erythrocytes (400 μl) were mixed with isopropanol, hexane and internal standard (20 μg 1,2-diheneicosanoyl-sn-glycero-3-phosphocholine dissolved into 40 μl chloroform), and were transmethylated with methanol and sulfuric acid. After incubating for 2.5 h at 80 °C and extracting two times by hexane, fatty acid methyl esters (FAMEs) were evaporated under nitrogen and then were redissolved in isoctane. FAMEs were analyzed by gas chromatography (Agilent 6890 N GC with flame ionization detector; SP-2560 capillary column: 100 m × 0.25 mm I.D. × 0.2 μm film; Supelco, Bellefonte, PA) with helium as carrier gas. The initial temperature was 90 °C and increased to 170, 175, 210, and 240 °C at different stage of chromatographic analysis. Quantification was conducted based on the peak area ratio of each fatty acid to the internal standard (1,2-diheneicosanoyl-sn-glycero-3-phosphocholine) and the concentration of internal standard. Relative amount of each fatty acid (% of total fatty acids) was quantified by expressing the area under each peak as a percentage of summed areas of all measured fatty acids (13 PUFAs, seven monounsaturated fatty acids [MUFAs], eight SFAs, and two TFAs), except for the internal standard [23]. Erythrocyte samples were analyzed in a random sequence. Quality control samples were made by a mixture of 500 ml erythrocyte samples from ~ 800 healthy volunteers aged 49.6 (9.9) from the Guizhou-Bijie Type 2 Diabetes Study [24] and aliquoted before analysis. They were inserted every 11 samples and processed by the same method as the tested samples to ensure repeatability. A total of ~ 990 quality control samples were analyzed and used to calculate the coefficients of variations (CVs). Overall, 10,563 (96.6%) of the 10,933 samples had valid fatty acids data after quality control.

Among all identified 30 fatty acids, there were eight n–6 PUFAs [18:2n–6 (linoleic acid), 18:3n–6 (γ-linolenic acid), 20:2n–6 (eicosadienoic acid), 20:3n–6 (dihomo-γ-linolenic acid), 20:4n–6 (arachidonic acid), 22:2n–6 (docosadienoic acid), 22:4n–6 (docosatetraenoic acid) and 22:5n–6 (docosapentaenoic acid)] and five n–3 PUFAs [18:3n–3 (α-linolenic acid), 20:3n–3 (eicosatrienoic acid), 20:5n–3 (eicosapentaenoic acid), 22:5n–3 (docosapentaenoic acid), and 22:6n–3 (docosahexaenoic acid)]. The CVs for most PUFAs were ≤ 10%, except for 22:2n–6 (11.0%), 20:5n–3 (10.6%), and 22:6n–3 (14.7%) (Supplementary Table 1). Two fatty acid ratios were calculated to estimate activity of desaturase enzymes, namely 20:4n–6/20:3n–6 ratio for delta-5 desaturase (D5D), and 18:3n–6/18:2n–6 ratio for delta-6 desaturase (D6D). The 20:3n-6/18:2n-6 ratio was also constructed to indicate the conversion of 18:2n–6 to 20:3n–6.

### Follow-up for stroke events

Information on stroke incidence was obtained periodically through linkage via a unique national identification number with the local death and disease (for cancer, stroke, ischemic heart disease and diabetes) registries, and with the universal national health insurance system, which covers any episodes of hospitalizations. In order to confirm survival status of the participants and to minimize losses to follow-up (currently < 1% since study entry at baseline), active follow-up was performed annually to check against local residential and administrative records. All stroke events reported from different sources between the date of 2013–14 resurvey (as baseline of the current analysis) and 1 Jan 2018 were checked and coded according to the International Classification of Diseases, 10th Revision (ICD-10), including total stroke (I60, I61, I63, and I64), IS (I63), and ICH (I61). Any hospital-reported cases of first stroke also underwent separate clinical adjudication, involving retrieval and review of original medical records and brain imaging reports (CT or MRI) by clinical specialists and > 90% of the reported first stroke cases were confirmed by brain imaging.

### Statistical analysis

Among the 10,563 participants who had fatty acids data, we excluded those who had been diagnosed with vascular diseases or diabetes prior to the 2013–14 resurvey (*n* = 2395) or had missing values for blood lipids (*n* = 9). After these exclusions, 8,159 participants remained in the main analyses (Supplementary Fig. 2).

Correlations between different PUFAs and of PUFAs with SBP, BMI, blood lipids, and dietary factors were evaluated by Spearman correlation coefficients (*r*). Cox regression was used to estimate hazard ratios (HRs) and 95% confidence intervals (CIs) for stroke events associated with quintiles of PUFAs, after adjustment for potential confounding factors including age (continuous variable), sex, study areas (10 regions), education attainment (no formal education, primary school, middle school, or high school and above), smoking status (never or occasional, ex-regular, current regular), alcohol drinking (never or occasional, ex-regular, current regular), family history of CVDs (yes/no), and physical activity (MET-hr/day). The linear trend of HRs over quintiles was assessed by χ^2^ test using these quintile numbers as continuous variables. The proportional hazards assumption in Cox regression was tested by the Schoenfeld residuals method and was not violated. In sensitivity analyses, HRs were further adjusted for the following circumstances, including the following: (1) potential mediators for CVD risks such as SBP, BMI, and LDL-C (model 1) in order to understand the underlying mechanisms linking PUFAs and stroke [25]; (2) fasting hours, dietary factors, and total n-3 PUFA (for n-6 PUFAs) or total n-6 PUFA (for n-3 PUFAs) (model 2) to understand the potential impacts of fasted/fed state, habitual dietary intake, and mutual influences of n-3 and n-6 PUFAs; and (3) total SFA and total MUFAs (model 3) to make sure the observed associations are independent of the other fatty acids.

For analyses involving more than two exposure categories, the floating absolute-risk method was applied. This method estimates standard errors and CIs for each category (including the reference category) using “floated” variances to provide appropriate variances to the log relative risk (i.e., HR in our analyses), without altering the value of the HRs [26]. Therefore, it enables comparisons among any two exposure categories for polychotomous risk factors. Potential nonlinear associations were also accessed by using restricted quadratic splines with three knots (5%, 50%, and 95%). Moreover, subgroup analysis by study areas was conducted for IS, while HRs were calculated per area-specific SD higher levels of erythrocyte PUFAs, and χ^2^ tests for heterogeneity were applied to the log HRs and their standard errors. Analyses were performed by R version 3.0 (http://R-project.org/). Two-sided *P* < 0.05 was considered as statistical significance.

## Results

Among the 8,159 participants, the mean (standard deviation, SD) age at the 2013–14 resurvey was 58.1 (9.9) years, 62% were females and mean (SD) BMI was 24.0 (3.4) kg/m^2^ (Table 1). Overall, male participants were more likely to attend formal school, and to be smokers and drinkers than females. Urban residents (47.2%) had lower levels of physical activity and consumption of coarse grain and refined grain, but higher levels of BMI, SBP, blood lipids, and red meat and fish intakes than their rural counterparts. The characteristics of the current subpopulation were comparable to those in overall CKB cohort (*n* = 512,715) and in the total population of the 2013–14 resurvey (*n* = 25,239) at the 2004–08 survey, except for a slightly higher percentage of urban residents by chance (Supplementary Table 2).

Compared with participants who did not develop stroke during follow-up, participants who developed any stroke and/or IS had higher levels of 18:3n–6, 20:3n–6, 22:5n–3, and ratios of 18:3n–6/18:2n–6 and 20:3n–6/18:2n–6, but lower 20:4n–6/20:3n–6 ratio (*P* < 0.05). Participants who developed ICH during follow-up had higher levels of 20:2n–6, 18:3n–3, 20:3n–3, 22:5n–3, but lower levels of 22:4n–6 and 22:6n–3 (*P* < 0.05) (Table 2). Erythrocyte PUFA levels also varied between females and males, as well as between urban and rural residents (Supplementary Table 3). Among the major PUFAs, the highest median level of 18:2n–6 was detected in participants from the northern city Harbin and the highest 20:5n–3 and 22:6n–3 levels in those living in the coastal city of Haikou (Supplementary Fig. 3). As expected, the 22:6n–3 level was higher in coastal residents than in inland residents. Residents from Gansu, an inland Province, had the highest levels of 18:3n–3 and 22:5n–3, but the lowest levels of 20:4n–6 and 22:6n–3.

Total n–6 PUFA and total n–3 PUFA were inversely correlated with each other (*r* = – 0.20, *P* < 0.001), while the correlations between individual PUFAs varied greatly, with the absolute value of correlation coefficients ranging from 0 to 0.73 (Supplementary Table 4). 18:3n-6 and 20:3n-6 were positively correlated with BMI (*r* = 0.12–0.13), whereas 20:3n-3 was inversely so (*r* = – 0.12, all *P* < 0.001; Supplementary Table 5). Among all PUFAs, 20:4n–6, 22:4n–6 and 22:5n–6 were positively correlated with LDL-C (*r* = 0.19–0.22); while 20:5n–3 was inversely correlated with LDL-C (*r* = – 0.12, all *P* < 0.001). Moreover, 20:4n–6 was positively correlated with red meat intake (*r* = 0.12); while 20:5n–3 and 22:6n–3 were positively correlated with fish intake (*r* = 0.18–0.21, all *P* < 0.001).

During a mean follow-up of 3.8 years (~ 30.6 thousand person-years), 412 incident stroke events were recorded, including 342 IS and 53 ICH cases. Among all n-6 PUFAs, higher 18:2n-6 level was associated with lower IS risk (adjusted HR = 0.69 [95% CI 0.53, 0.90] for top versus bottom quintile, *P*_trend_ = 0.027; Table 3), but higher ICH risk (2.33 [1.41, 3.82], *P*_trend_ = 0.007). In addition, 20:3n–6 showed positive associations with total stroke, with HRs comparing extreme quintiles being 1.51 (1.23, 1.84, *P*_trend_ = 0.001). This association was mainly driven by a positive association with IS (HR 1.64 [1.32, 2.04], *P*_trend_ < 0.001), with no significant association with ICH (1.02 [0.55, 1.89], *P*_trend_ = 0.96) observed. Further adjustment for SBP, BMI, LDL-C, dietary factors, total n-3 PUFA, SFA, and MUFA did not materially altered these associations (Supplementary Table 6). Moreover, 22:2n–6 level was inversely associated with risk of IS (0.73 [0.56, 0.93], *P*_trend_ = 0.038, Table 3), while higher 22:5n–6 level was associated with lower ICH risk (0.46 [0.20, 1.07], *P*_trend_ = 0.032).

**Fig. 1:**
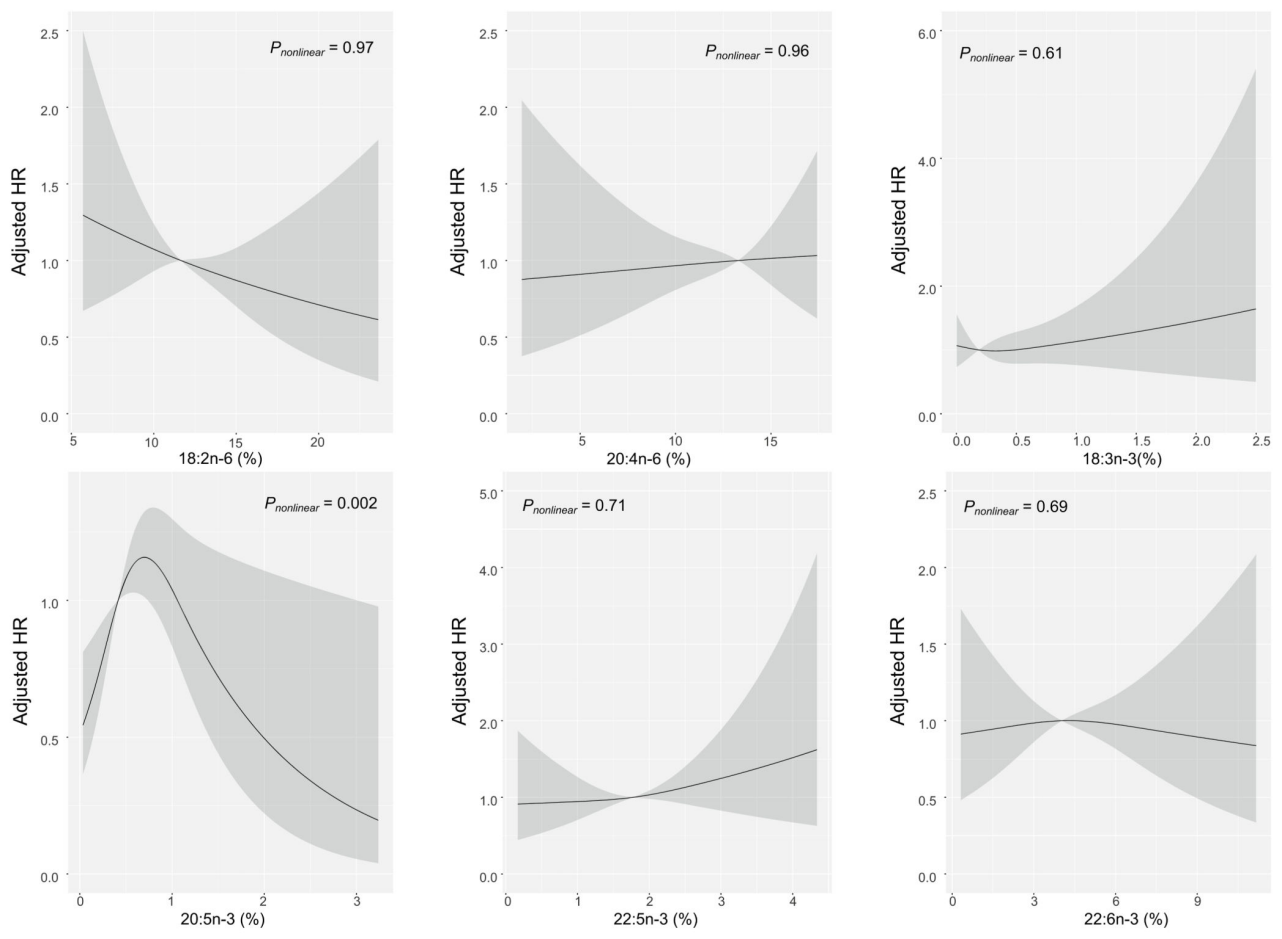
Associations of erythrocyte PUFAs with risk of ischemic stroke by restricted cubic splines from Cox proportional hazards models. Model was adjusted for age, sex, study areas, education, smoking, alcohol drinking, family history of cardiovascular diseases, and physical activity. The solid lines represent the HRs, and the shaded areas represent 95% CIs, relative to the reference level (50th percentile)

Among n–3 PUFAs, higher level of 20:5n–3 was associated with higher risk of total stroke and IS (Table 4), with the highest HR observed in the fourth quintile (1.35 [1.09, 1.67], *P*_trend_ = 0.029) and 1.51 [1.21, 1.89, *P*_trend_ = 0.036], respectively), compared with the bottom quintile. After further adjustment for SBP, BMI, and LDL-C, these associations became non-significant (Supplementary Table 7), which may be partially mediated through BMI and SBP (Supplementary Table 8). Restricted cubic splines analysis also detected inverted-*U* shape curve associations of 20:5n–3 with IS (*P*_nonlinear_ = 0.002, Fig. 1) and total stroke (*P*_nonlinear_ = 0.008, Supplementary Fig. 4) with a threshold at 20:5n–3 level of 0.70%, which remained similar after further adjustment (data not show). Moreover, we observed a significant positive association between 20:3n–3 and ICH, with HR in the top quintile being 6.17 [3.43, 11.1] in comparison with bottom quintile, *P*_trend_ = 0.005; Table 4).

For fatty acids ratios, the 20:3n–6/18:2n–6 ratio was positively associated with risk of IS (but not ICH), with HRs being 1.84 (1.47, 2.32) when comparing the fifth with the first quintile (Supplementary Table 9, model 1, *P*_trend_ < 0.001). In contrast, the 20:4n-6/20:3n-6 ratio was inversely associated with IS (0.70 [0.53, 0.92], *P*_trend_ = 0.010) (not ICH), but the associations attenuated significantly after further adjustments for SBP, BMI, and LDL-C. The associations with total stroke were very similar to those with IS. In subgroup analysis, no significant heterogeneity across study areas was observed in the abovementioned associations between PUFAs and IS risk (Supplementary Table 10, *P*_heterogeneity_ > 0.05).

## Discussion

In this large prospective study of relatively lean Chinese adults, the levels of erythrocyte PUFAs varied by geographic areas and were associated with certain dietary factors. Among the 13 PUFAs analyzed, 18:2n–6 showed significant association with both IS and ICH (inversely with IS and positively with ICH), while 20:3n–6 showing significant positive associations with IS but not ICH. Moreover, inverted-*U* shape curve associations were observed of 20:5n–3 with IS and total stroke.

According to nationwide dietary surveys, total dietary intake of PUFAs was high (8.6% daily energy) but marine n–3 PUFA intake was low (3.6 mg/d) among Chinse adults [27], compared with Western and Japanese populations [28]. Though the data of dietary PUFA intake were not available in the present study, levels of erythrocyte 18:2n–6, 20:4n–6, and 18:3n–3 were comparable with those in Western studies; however, the levels of 20:5n–3 (median 0.42 [0.27; 0.64]) and 22:6n–3 (median 4.03 [3.09; 5.27]) were relatively lower [16, 29, 30]. Notably, within China, there were great geographical variations in dietary patterns [17], which might be a reason for the large regional difference of erythrocyte n–6 and n–3 PUFAs observed in our study. However, no significant heterogeneity across study areas was observed in the associations between PUFAs and IS.

While more prospective studies have reported on the relationship of plasma/serum PUFAs with CVDs, to date only four prospective studies (all among Western populations) have examined the relations of erythrocyte PUFAs with stroke [13–16, 31–37]. Moreover, previous studies tended to focused mainly on IS, and typically involved modest number of cases (mostly < 300). Individually, the associations of PUFAs with stroke were inconsistent, with some studies showing inverse association of 18:2n–6 or marine n–3 PUFAs with IS and/or total stroke [13–15, 31, 36], while others reporting null associations [32, 34, 35]. A pooled analysis of 21 cohort studies (19 among Western populations) assessing levels of 18:2n-6 and 20:4n–6 in different lipid fractions in relation to IS risk has found a significant inverse association of 18:2n–6 with incident IS [16]. In East Asian populations who had higher stroke rates and substantially differed dietary patterns than in Western populations, evidence linking blood levels of PUFAs with stroke risk is very limited [36, 37]. In Japan, a nested case–6control study involving < 200 stroke cases showed that per SD higher serum 18:2n–6 level was associated with 28–34% lower risks of total stroke and IS, but no clear association with ICH [36]. Notably, our study also extended the previously reported positive associations of 20:3n–6 with risks of diabetes and heart diseases [38, 39] to total stroke and IS, but the underlying mechanisms merits further investigation. Moreover, our study did not detect association between 20:4n–6 and stroke risk, which might be due to the fact that 20:4n-6 is precursors for both pro-inflammation and anti-inflammation metabolites [7].

Taken together, previous studies of PUFAs have only included a total of ~ 230 cases of ICH [33–36]. In contrast to the significant positive associtions observed in the present study, previous studies generally did not observe any apparent association of 18:2n–6 with incident ICH. The mechanisms underlying the contrasting associations of 18:2n–6 with IS and ICH were not clear and may reflect the potential effects of 18:2n–6 on lowering blood LDL-C and platelet aggregation [40, 41], which would be beneficial for IS but harmful for ICH [42, 43]. Notably, vegetarians were reported to have higher rates of hemorrhagic stroke than meat eaters in British population [44], which supported our finding since 18:2n–6 is largely derived from plant oils.

Among n–3 PUFAs, 20:5n–3 was positively associated with IS, which was partially mediated through BMI and SBP. Our result was in accordance to the null association between 20:5n–3 and IS reported in several US cohort studies with adjustment of BMI and/or SBP (1917 cases in total) [13, 31]. On the other hand, a Finnish study reported a positive association between serum marine n–3 PUFAs and IS risk (153 cases), although only in subjects having higher hair mercury [33]. Thus, it needs to be clarified whether environment pollutants in marine products could mask the potential associations of marine n–3 PUFAs with stroke risks. Interestingly, a threshold of 20:5n–3 level at 0.7% (higher than ~ 80% of our participants) was detected by the restricted cubic splines analysis, with inverse associations observed above this level. Given the low consumption of marine n–3 PUFAs among Chinese [27] and large regional variation in n–3 PUFA levels, it would be of importance to identify an ideal 20:5n–3 level for cardio-metabolic health.

The present study has several major strengths, besides its prospective design. First, we measured erythrocyte PUFAs which reflect relatively long-term average levels of PUFAs and are less susceptible to biological variations and measurement errors (e.g. reporting errors). Second, the statistical analyses had controlled for a variety of potential confounding factors, limiting the impacts of residual confounding. Third, we examined the associations with different subtypes of stroke separately, which is important given the different etiology of IS and ICH. Fourth, the large variation in PUFA levels allowed us to detect the nonlinear association of 20:5n–3 with stroke risks. However, the study also has limitations. First, owing to relatively short follow-up time, we were unable to fully explore potential reverse causality by excluding the first few years of follow-up. However, such bias would have been controlled to a large extent by excluding those with prior CVD and diabetes from the main analyses. Second, small number of ICH cases limited the statistical power. Third, the dietary questionnaire only covered some of major food groups, so we were unable to compare findings based on nutrient biomarkers with self-reported nutrient intakes. Fourth, we did not correct for multiple testing because our study is hypothesis testing instead of hypothesis generating. Those significant results observed in our study could be a chance finding. Last, given the observational nature of the study and the possibility of residual confounding, causality cannot be fully confirmed.

In summary, the present study provided new evidence for several PUFAs with different types of stroke among Chinese adults, including the contrasting associations of 18:2n–6 with risks of IS (inverse) and ICH (positive), and the positive associations of 20:3n–6 with total stroke and IS. The associations of 20:5n–3 with IS and total stroke appeared to be nonlinear with a threshold at 0.7%. Further larger studies are needed to elucidate these associations.

## Supplementary Material

The online version contains supplementary material available at https://doi.org/10.1007/s00394-022-02879-y.

Supplementary information

## Figures and Tables

**Table 1:** Characteristics of the study participants in 2013–14

Characteristics	Males		Females		Total (*n* = 8159)
	Rural (*n* = 1672)	Urban (*n* = 1431)	Rural (*n* = 2633)	Urban (*n* = 2423)	
Age, years	58.9 ± 10.0	59.1 ± 10.2	57.1 ± 9.7	58.2 ± 9.8	58.1 ± 9.9
No formal schooling, %	12.5	5.6	30.2	18.9	18.9
High household income, %	35.5	60.6	33.5	49.0	43.3
Ever regular smoker, %	70.7	63.6	2.6	1.2	26.8
Ever regular drinker, %	37.5	41.1	3.5	3.2	17.0
Physical activity, MET-hr/day	21.0 ± 16.3	18.3 ± 13.5	19.1 ± 13.1	17.6 ± 12.6	18.9 ± 13.8
*Daily dietary intake, g*
Refined grain	365.0 ± 156.4	334.8 ± 143.9	295.0 ± 119.6	260.3 ± 114. 5	306.0 ± 136.6
Coarse grain	43.0 ± 80.0	28.0 ± 46.9	34.0 ± 72.2	30.9 ± 47.9	33.9 ± 63.9
Red meat	61.7 ± 59.2	73.6 ± 63.0	43.7 ± 46.6	53.6 ± 42.5	55.6 ± 52.5
Fish	10.3 ± 21.3	56.3 ± 74.6	7.2 ± 16.6	44.7 ± 56.8	27.6 ± 50.4
BMI, kg/m^2^	23.3 ± 3.3	24.5 ± 3.2	23.8 ± 3.4	24.4 ± 3.4	24.0 ± 3.4
SBP, mmHg	133.4 ± 19.5	138.4 ± 19.3	133.3 ± 21.2	135.4 ± 20.6	134.8 ± 20.4
LDL-C, mmol/l	1.61 ± 0.96	2.16 ± 1.40	1.66 ± 0.96	2.29 ± 1.47	1.93 ± 1.25
HDL-C, mmol/l	1.32 ± 0.51	1.33 ± 0.64	1.43 ± 0.47	1.55 ± 0.70	1.43 ± 0.59
Triglycerides, mmol/l	1.43 ± 1.16	1.83 ± 1.78	1.62 ± 1.02	1.81 ± 1.85	1.67 ± 1.48
Total cholesterol, mmol/l	3.53 ± 1.19	4.16 ± 1.67	3.78 ± 1.23	4.52 ± 1.86	4.01 ± 1.56

Values were either percentage or mean ± SD Abbreviations: *BMI* body mass index, *HDL-C* high-density lipoprotein cholesterol, *LDL-C* low-density lipoprotein cholesterol, *MET* metabolic equivalent, *SBP* systolic blood pressure

a Annual household income ≥ 50,000 yuan

**Table 2:** Median concentrations [Q1;Q3] of erythrocyte PUFAs of study participants by status of incident stroke types

PUFAs	Total stroke (*n* = 412)	IS (*n* = 342)	ICH (*n* = 53)	None-stroke (*n* = 7747)	Total population (*n* = 8159)
*Total n-6 PUFAs, %*	30.5 [28.3;32.8]	30.4 [28.3;32.7]	30.3 [27.6;32.4]	30.4 [28.4;32.4]	30.4 [28.4;32.4]
18:2n–6 (linoleic acid)	11.7 [10.3;13.1]	11.7 [10.3;13.0]	12.2 [10.3;13.8]	11.7 [10.5;13.0]	11.7 [10.5;13.0]
18:3n–6 (γ-linolenic acid)	0.057 [0.037;0.089]*	0.057 [0.037;0.089]*	0.058 [0.037;0.095]	0.054 [0.034;0.085]	0.054 [0.034 0.085]
20:2n–6 (eicosadienoic acid)	0.40 [0.36;0.45]	0.40 [0.36;0.45]	0.43 [0.37;0.48]*	0.40 [0.35;0.45]	0.40 [0.35;0.45]
20:3n–6 (dihomo-γ-linolenic acid)	1.36 [1.17;1.59]*	1.38 [1.18;1.60]*	1.31 [1.11;1.52]	1.29 [1.10;1.51]	1.29 [1.11;1.51]
20:4n–6 (arachidonic acid)	13.2 [12.1;14.4]	13.3 [12.2;14.5]	12.9 [11.5;14.1]	13.3 [12.1;14.3]	13.3 [12.1;14.3]
22:2n–6 (docosadienoic acid)	0.080 [0.062;0.103]	0.080 [0.062;0.102]	0.088 [0.061;0.110]	0.081 [0.065;0.102]	0.081 [0.065;0.102]
22:4n–6 (docosatetraenoic acid)	2.67 [2.12;3.27]	2.68 [2.15;3.26]	2.48 [1.79;2.97]*	2.69 [2.14;3.22]	2.69 [2.14;3.22]
22:5n–6 (docosapentaenoic acid)	0.56 [0.41;0.79]	0.57 [0.42;0.78]	0.49 [0.35;0.78]	0.56 [0.42;0.73]	0.56 [0.42;0.73]
*Total n-3 PUFAs, %*	6.80 [5.50;8.09]	6.85 [5.56;8.11]	6.33 [5.43;8.00]	6.64 [5.56;8.04]	6.65 [5.55;8.04]
18:3n–3 (α;linolenic acid)	0.19 [0.12;0.31]	0.18 [0.12;0.30]	0.24 [0.14;0.42]*	0.19 [0.12;0.28]	0.19 [0.12;0.28]
20:3n–3 (eicosatrienoic acid)	0.048 [0.034;0.070]	0.048 [0.034;0.068]	0.065 [0.044;0.100]*	0.048 [0.034;0.068]	0.048 [0.034;0.068]
20:5n–3 (eicosapentaenoic acid)	0.44 [0.26;0.67]	0.45 [0.27;0.67]	0.41 [0.28;0.70]	0.41 [0.27;0.64]	0.42 [0.27;0.64]
22:5n–3 (docosapentaenoic acid)	1.82 [1.53;2.14]*	1.83 [1.52;2.13]	1.85 [1.62;2.26]*	1.77 [1.49;2.05]	1.77 [1.49;2.05]
22:6n–3 (docosahexaenoic acid)	3.98 [3.05;5.30]	4.08 [3.11;5.37]	3.49 [2.71;4.52]*	4.03 [3.10;5.27]	4.03 [3.09;5.27]
*Fatty acids ratio*					
18:3n–6/18:2n–6	0.0047 [0.0031;0.0084]*	0.0048 [0.0032;0.0085]*	0.0047 [0.0029;0.0089]	0.0046 [0.0028;0.0073]	0.0046 [0.0028;0.0074]
20:3n–6/18:2n–6	0.12 [0.10;0.14]*	0.12 [0.10;0.14]*	0.11 [0.09;0.13]	0.11 [0.09;0.13]	0.110 [0.091;0.133]
20:4n–6/20:3n–6	9.5 [8.1;11.6]*	9.6 [8.1;11.5]*	9.3 [8.3;11.6]	10.0 [8.2;11.9]	10.0 [8.2;11.9]
Total n–6/total n–3	4.51 [3.74;5.69]	4.46 [3.73;5.67]	4.70 [3.79;5.59]	4.60 [3.70;5.66]	4.60 [3.70;5.66]

Abbreviations: *ICH* intracerebral hemorrhage, *IS* ischemic stroke, *PUFA* polyunsaturated fatty acid

* *P* < 0.05 when comparing stroke cases with non-stroke participants by Mann–Whitney *U* test

**Table 3:** Adjusted hazard ratios (95% CI) for incident stroke events associated with n-6 PUFA quintiles

	Q1	Q2	Q3	Q4	Q5	*P*_trend_
*Total stroke*						
18:2n–6	1.00 (0.80,1.26)	0.94 (0.75,1.17)	0.82 (0.65,1.04)	0.89 (0.72,1.11)	0.87 (0.70,1.09)	0.38
18:3n–6	1.00 (0.77,1.30)	1.29 (1.03,1.61)	1.42 (1.15,1.76)	1.42 (1.15,1.76)	1.38 (1.11,1.71)	0.084
20:2n–6	1.00 (0.78,1.28)	0.90 (0.71,1.14)	0.86 (0.68,1.07)	1.01 (0.81,1.24)	0.99 (0.79,1.22)	0.76
20:3n–6	1.00 (0.77,1.29)	0.94 (0.73,1.19)	1.05 (0.84,1.32)	1.25 (1.02,1.54)	1.51 (1.23,1.84)	0.001
20:4n–6	1.00 (0.79,1.27)	0.89 (0.71,1.11)	0.99 (0.80,1.23)	0.75 (0.59,0.97)	0.94 (0.74,1.18)	0.47
22:2n–6	1.00 (0.80,1.26)	0.82 (0.65,1.03)	0.80 (0.63,1.01)	0.77 (0.61,0.97)	0.78 (0.62,0.98)	0.13
22:4n–6	1.00 (0.78,1.29)	1.22 (0.99,1.51)	1.04 (0.83,1.31)	0.91 (0.71,1.17)	0.93 (0.70,1.23)	0.23
22:5n–6	1.00 (0.78,1.28)	0.90 (0.72,1.14)	0.82 (0.64,1.04)	0.82 (0.64,1.04)	0.85 (0.64,1.13)	0.27
Total n–6	1.00 (0.78,1.29)	0.87 (0.69,1.10)	0.89 (0.71,1.13)	0.73 (0.57,0.93)	0.91 (0.70,1.18)	0.35
*Ischemic stroke*						
18:2n–6	1.00 (0.78,1.29)	1.01 (0.80,1.27)	0.82 (0.64,1.05)	0.84 (0.66,1.07)	0.69 (0.53,0.90)	0.027
18:3n–6	1.00 (0.75,1.33)	1.16 (0.90,1.49)	1.39 (1.11,1.75)	1.41 (1.12,1.77)	1.37 (1.08,1.73)	0.061
20:2n–6	1.00 (0.76,1.31)	0.93 (0.72,1.20)	0.88 (0.68,1.12)	1.09 (0.87,1.37)	0.93 (0.72,1.19)	0.91
20:3n–6	1.00 (0.76,1.32)	0.86 (0.65,1.13)	1.09 (0.85,1.39)	1.33 (1.06,1.66)	1.64 (1.32,2.04)	< 0.001
20:4n–6	1.00 (0.77,1.30)	0.94 (0.73,1.21)	1.11 (0.88,1.39)	0.84 (0.64,1.10)	1.06 (0.82,1.37)	0.95
22:2n–6	1.00 (0.78,1.28)	0.87 (0.68,1.10)	0.79 (0.62,1.02)	0.71 (0.55,0.92)	0.73 (0.56,0.93)	0.038
22:4n–6	1.00 (0.75,1.33)	1.40 (1.11,1.76)	1.17 (0.91,1.50)	1.01 (0.77,1.32)	1.05 (0.77,1.43)	0.48
22:5n–6	1.00 (0.76,1.32)	0.95 (0.74,1.22)	0.86 (0.66,1.13)	0.94 (0.73,1.22)	0.95 (0.69,1.31)	0.80
Total n–6	1.00 (0.76,1.32)	0.87 (0.67,1.12)	0.98 (0.76,1.25)	0.69 (0.53,0.90)	0.85 (0.64,1.13)	0.21
*Intracerebral hemorrhage*						
18:2n–6	1.00 (0.52,1.93)	0.57 (0.24,1.37)	0.97 (0.48,1.94)	1.46 (0.81,2.64)	2.33 (1.41,3.82)	0.007
18:3n–6	1.00 (0.46,2.19)	1.57 (0.88,2.80)	1.14 (0.59,2.19)	1.48 (0.82,2.68)	1.39 (0.77,2.53)	0.69
20:2n–6	1.00 (0.49,2.04)	0.73 (0.34,1.53)	0.69 (0.33,1.45)	0.84 (0.43,1.61)	1.54 (0.94,2.54)	0.17
20:3n–6	1.00 (0.52,1.92)	1.10 (0.61,2.00)	0.70 (0.33,1.46)	1.11 (0.62,1.96)	1.02 (0.55,1.89)	0.96
20:4n–6	1.00 (0.55,1.81)	0.80 (0.44,1.44)	0.78 (0.43,1.42)	0.40 (0.16,0.97)	0.60 (0.29,1.26)	0.15
22:2n–6	1.00 (0.52,1.92)	0.36 (0.13,0.95)	0.85 (0.44,1.64)	1.11 (0.63,1.96)	1.32 (0.77,2.25)	0.20
22:4n–6	1.00 (0.54,1.85)	0.66 (0.36,1.21)	0.47 (0.23,0.96)	0.46 (0.21,0.99)	0.43 (0.18,1.03)	0.060
22:5n–6	1.00 (0.54,1.84)	0.66 (0.36,1.24)	0.40 (0.18,0.89)	0.29 (0.12,0.72)	0.46 (0.20,1.07)	0.032
Total n–6	1.00 (0.52,1.92)	0.82 (0.43,1.55)	0.49 (0.20,1.19)	1.21 (0.65,2.24)	1.32 (0.63,2.74)	0.40

Model was adjusted for age, sex, study areas, education, smoking, alcohol drinking, family history of cardiovascular diseases, and physical activity Cox regression was used to estimate hazard ratios and 95% confidence intervals. For analyses involving more than two exposure categories, the floating absolute-risk method was applied to provide 95% CI for each category Abbreviations: *CI* confidence interval, *SD* standard deviation, *PUFA* polyunsaturated fatty acid

**Table 4:** Adjusted hazard ratios (95% CI) for incident stroke events associated with n-3 PUFA quintiles

	Q1	Q2	Q3	Q4	Q5	*P*_trend_
*Total stroke*						
18:3n–3	1.00 (0.77,1.29)	0.96 (0.76,1.20)	0.89 (0.71,1.11)	0.69 (0.53,0.90)	1.15 (0.89,1.50)	0.89
20:3n–3	1.00 (0.78,1.28)	0.95 (0.76,1.19)	1.03 (0.83,1.28)	0.90 (0.72,1.13)	1.13 (0.87,1.46)	0.68
20:5n–3	1.00 (0.77,1.30)	0.75 (0.58,0.97)	1.05 (0.84,1.32)	1.35 (1.09,1.67)	1.12 (0.89,1.42)	0.029
22:5n–3	1.00 (0.78,1.28)	0.94 (0.74,1.19)	1.35 (1.09,1.66)	0.93 (0.73,1.19)	1.37 (1.10,1.69)	0.073
22:6n–3	1.00 (0.77,1.29)	0.95 (0.75,1.19)	0.90 (0.71,1.13)	1.13 (0.89,1.43)	0.95 (0.72,1.27)	0.76
Total n–3	1.00 (0.79,1.26)	0.96 (0.75,1.22)	0.90 (0.71,1.14)	1.35 (1.09,1.68)	1.08 (0.83,1.42)	0.14
*Ischemic stroke*						
18:3n–3	1.00 (0.75,1.32)	0.97 (0.76,1.24)	0.94 (0.74,1.20)	0.70 (0.53,0.94)	1.11 (0.83,1.49)	0.75
20:3n–3	1.00 (0.77,1.30)	0.89 (0.69,1.13)	1.02 (0.81,1.29)	0.80 (0.62,1.04)	1.02 (0.76,1.36)	0.84
20:5n–3	1.00 (0.75,1.34)	0.81 (0.61,1.07)	1.03 (0.80,1.33)	1.51 (1.21,1.89)	1.10 (0.85,1.43)	0.036
22:5n–3	1.00 (0.77,1.31)	0.85 (0.65,1.11)	1.31 (1.05,1.65)	0.91 (0.70,1.19)	1.31 (1.04,1.65)	0.11
22:6n–3	1.00 (0.74,1.34)	1.05 (0.81,1.36)	0.98 (0.76,1.26)	1.22 (0.95,1.58)	1.04 (0.76,1.41)	0.56
Total n–3	1.00 (0.77,1.29)	0.86 (0.65,1.14)	0.99 (0.77,1.27)	1.34 (1.06,1.70)	1.02 (0.76,1.37)	0.20
*Intracerebral hemorrhage*						
18:3n–3	1.00 (0.43,2.31)	1.08 (0.52,2.23)	1.01 (0.50,2.01)	0.88 (0.41,1.89)	1.70 (0.86,3.37)	0.41
20:3n–3	1.00 (0.30,3.32)	2.61 (1.18,5.75)	3.28 (1.61,6.68)	4.14 (2.38,7.21)	6.17 (3.43,11.1)	0.005
20:5n–3	1.00 (0.45,2.22)	1.18 (0.58,2.39)	2.24 (1.31,3.82)	1.24 (0.62,2.52)	1.75 (0.93,3.29)	0.36
22:5n–3	1.00 (0.41,2.46)	2.41 (1.31,4.46)	2.47 (1.37,4.47)	1.58 (0.75,3.33)	2.54 (1.42,4.54)	0.37
22:6n–3	1.00 (0.54,1.84)	0.77 (0.40,1.48)	1.09 (0.58,2.06)	1.13 (0.51,2.52)	0.74 (0.27,2.01)	0.99
Total n–3	1.00 (0.51,1.97)	2.02 (1.16,3.52)	0.54 (0.20,1.43)	2.05 (1.12,3.75)	2.00 (0.95,4.24)	0.25

Model was adjusted for age, sex, study areas, education, smoking, alcohol drinking, family history of cardiovascular diseases, and physical activity Cox regression was used to estimate hazard ratios and 95% confidence interval For analyses involving more than two exposure categories, the floating absolute-risk method was applied to provide 95% CI for each category Abbreviations: *CI* confidence interval, *SD* standard deviation, *PUFA* polyunsaturated fatty acid

## Data Availability

Data will be made available upon request pending application and approval. Details are available from www.ckbiobank.org/site/Data+Access.
