# Supplementary material for: Associations of erythrocyte polyunsaturated fatty acids with incidence of stroke and stroke types in adult Chinese: a prospective study of over 8000 individuals
Source: Eur J Nutr. Author manuscript; Available in PMC 2022 Sep 7. (PMC9363313; doi:10.1007/s00394-022-02879-y)
Supplement: Supplementary information [file EMS146000-supplement-Supplementary_information.docx]

**Associations of erythrocyte polyunsaturated fatty acids with incidence of stroke and stroke types in adult Chinese: a prospective study of over 8,000 individuals**

**European Journal of Nutrition**

Liang Sun^*^, Huaidong Du^*^, Geng Zong, Yu Guo, Yan Chen, Yiping Chen, Huiyong Yin, Pei Pei, Ling Yang, Qianqian Chu, Canqing Yu, Yixue Li, Jun Lv, He Zheng, Puchen Zhou, Junshi Chen, Liming Li^†^, Zhengming Chen^†^, Xu Lin^†^, on behalf of the China Kadoorie Biobank Collaborative Group

*^*^ Joint first authors; † Joint senior authors*

**Address for correspondence**

Xu Lin, MD, PhD, Shanghai Institute of Nutrition and Health, Chinese Academy of Sciences, 320 Yue-yang Rd., Shanghai, 200031, China; email: [xlin@sibs.ac.cn](mailto:xlin@sibs.ac.cn); or

Zhengming Chen, MBBS, DPhil, Big Data Institute Building, Old Road Campus, University of Oxford, Oxford OX3 7LF, UK; email: [zhengming.chen@ndph.ox.ac.uk](mailto:zhengming.chen@ndph.ox.ac.uk)

**Supplementary Information**

1. Members of the China Kadoorie Biobank Collaborative Group
2. Supplementary Table 1. Measurement of erythrocyte PUFAs by gas chromatography with flame ionization detector among 10,563 participants.
3. Supplementary Table 2. Comparisons of characteristics of participants in the overall CKB cohort, 2013-14 resurvey population, and the subpopulation in current analysis at the 2004-08 baseline survey.
4. Supplementary Table 3. Median concentrations [Q1;Q3] of erythrocyte PUFAs of study participants by sex and residence (rural/urban).
5. Supplementary Table 4. Spearman correlations of different PUFAs.
6. Supplementary Table 5. Spearman correlations of PUFAs with cardio-metabolic traits and daily dietary intake.
7. Supplementary Table 6. Adjusted hazard ratios (95% CI) for stroke events associated with n-6 PUFA quintiles in sensitivity analysis.
8. Supplementary Table 7. Adjusted hazard ratios (95% CI) for stroke events associated with n-3 PUFA quintiles in sensitivity analysis.
9. Supplementary Table 8. Adjusted hazard ratios (95% CI) for stroke events associated with 20:5n-3 quintiles.
10. Supplementary Table 9. Adjusted hazard ratios (95% CI) for stroke events associated with PUFA ratio quintiles.
11. Supplementary Table 10. Associations of PUFAs with risks of ischemic stroke in subgroups analysis by study areas.
12. Supplementary Fig. 1. The metabolism of n-3 and n-6 PUFAs in human.
13. Supplementary Fig. 2. Flow chart of participant selection.
14. Supplementary Fig. 3. Median concentrations [Q1;Q3] of major PUFAs by study areas.
15. Supplementary Fig. 4. Associations of erythrocyte PUFAs with risk of total stroke by restricted cubic splines from Cox proportional hazards models.

**Members of the China Kadoorie Biobank Collaborative Group**

*International Steering Committee:* Junshi Chen, Zhengming Chen (PI), Robert Clarke, Rory Collins, Yu Guo, Liming Li (PI), Jun Lv, Richard Peto, Robin Walters.

*International Co-ordinating Centre, Oxford:* Daniel Avery, Ruth Boxall, Derrick Bennett, Yumei Chang, Yiping Chen, Zhengming Chen, Robert Clarke, Huaidong Du, Simon Gilbert, Alex Hacker, Michael Holmes, Andri Iona, Christiana Kartsonaki, Rene Kerosi, Garry Lancaster, Kuang Lin, John McDonnell, Iona Millwood, Qunhua Nie, Richard Peto, Jayakrishnan Radhakrishnan, Paul Ryder, Sam Sansome, Dan Schmidt, Rajani Sohoni, Becky Stevens, Iain Turnbull, Robin Walters, Jenny Wang, Lin Wang, Neil Wright, Ling Yang, Xiaoming Yang.

*National Co-ordinating Centre, Beijing:* Zheng Bian, Ge Chen, Yu Guo, Xiao Han, Can Hou, Chao Liu, Pei Pei, Shuzhen Qu, Yunlong Tan, Canqing Yu.

*10 Regional Co-ordinating Centres:*

*Qingdao* Qingdao CDC: Zengchang Pang, Ruqin Gao, Shanpeng Li, Shaojie Wang, Yongmei Liu, Ranran Du, Yajing Zang, Liang Cheng, Xiaocao Tian, Hua Zhang, Yaoming Zhai, Feng Ning, Xiaohui Sun, Feifei Li. Licang CDC: Silu Lv, Junzheng Wang, Wei Hou.

*Harbin* Heilongjiang Provincial CDC: Mingyuan Zeng, Ge Jiang, Xue Zhou. Nangang CDC: Liqiu Yang, Hui He, Bo Yu, Yanjie Li, Qinai Xu, Quan Kang, Ziyan Guo.

*Haikou* Hainan Provincial CDC: Dan Wang, Ximin Hu, Jinyan Chen, Yan Fu, Zhenwang Fu, Xiaohuan Wang. Meilan CDC: Min Weng, Zhendong Guo, Shukuan Wu, Yilei Li, Huimei Li, Zhifang Fu.

*Suzhou* Jiangsu Provincial CDC: Ming Wu, Yonglin Zhou, Jinyi Zhou, Ran Tao, Jie Yang, Jian Su. Suzhou CDC: Fang Liu, Jun Zhang, Yihe Hu, Yan Lu, Liangcai Ma, Aiyu Tang, Shuo Zhang, Jianrong Jin, Jingchao Liu.

*Liuzhou* Guangxi Provincial CDC: Zhenzhu Tang, Naying Chen, Ying Huang. Liuzhou CDC: Mingqiang Li, Jinhuai Meng, Rong Pan, Qilian Jiang, Jian Lan, Yun Liu, Liuping Wei, Liyuan Zhou, Ningyu Chen, Ping Wang, Fanwen Meng, Yulu Qin, Sisi Wang.

*Sichuan* Sichuan Provincial CDC: Xianping Wu, Ningmei Zhang, Xiaofang Chen, Weiwei Zhou. Pengzhou CDC: Guojin Luo, Jianguo Li, Xiaofang Chen, Xunfu Zhong, Jiaqiu Liu, Qiang Sun.

*Gansu* Gansu Provincial CDC: Pengfei Ge, Xiaolan Ren, Caixia Dong. Maiji CDC: Hui Zhang, Enke Mao, Xiaoping Wang, Tao Wang, Xi Zhang.

*Henan* Henan Provincial CDC: Ding Zhang, Gang Zhou, Shixian Feng, Liang Chang, Lei Fan. Huixian CDC: Yulian Gao, Tianyou He, Huarong Sun, Pan He, Chen Hu, Xukui Zhang, Pan He, Huifang Wu.

*Zhejiang* Zhejiang Provincial CDC: Min Yu, Ruying Hu, Hao Wang. Tongxiang CDC: Yijian Qian, Chunmei Wang, Kaixu Xie, Lingli Chen, Yidan Zhang, Dongxia Pan, Qijun Gu.

*Hunan* Hunan Provincial CDC: Yuelong Huang, Biyun Chen, Li Yin, Huilin Liu, Zhongxi Fu, Qiaohua Xu. Liuyang CDC: Xin Xu, Hao Zhang, Huajun Long, Xianzhi Li, Libo Zhang, Zhe Qiu.

**Supplementary Table 1. Measurement of erythrocyte PUFAs by gas chromatography with flame ionization detector among 10,563 participants.**

| **PUFAs** | **Retention time (min)** | **Median**  **(Q1,Q3) (%)** | **CV (%)** |
| --- | --- | --- | --- |
| 18:2n-6 (linoleic acid) | 44.68 | 11.7 (10.4,13.1) | 3.46 |
| 18:3n-6 (γ-linolenic acid) | 47.63 | 0.05 (0.03,0.09) | 7.74 |
| 20:2n-6 (eicosadienoic acid) | 53.01 | 0.40 (0.35,0.45) | 8.43 |
| 20:3n-6 (dihomo-γ-linolenic acid) | 56.35 | 1.29 (1.11,1.52) | 5.22 |
| 20:4n-6 (arachidonic acid) | 58.60 | 13.2 (12.0,14.3) | 4.07 |
| 22:2n-6 (docosadienoic acid) | 61.11 | 0.08 (0.06,0.10) | 11.0 |
| 22:4n-6 (docosatetraenoic acid) | 66.04 | 2.67 (2.12,3.21) | 8.03 |
| 22:5n-6 (docosapentaenoic acid) | 67.76 | 0.55 (0.41,0.73) | 8.77 |
| 18:3n-3 (α-linolenic acid) | 49.29 | 0.18 (0.12,0.28) | 6.51 |
| 20:3n-3 (eicosatrienoic acid) | 58.00 | 0.05 (0.03,0.07) | 9.07 |
| 20:5n-3 (eicosapentaenoic acid) | 62.78 | 0.42 (0.27,0.65) | 10.6 |
| 22:5n-3 (docosapentaenoic acid) | 70.22 | 1.77 (1.49,2.06) | 6.29 |
| 22:6n-3 (docosahexaenoic acid) | 72.12 | 4.08 (3.11,5.34) | 14.7 |

Abbreviations: PUFA, polyunsaturated fatty acid.

**Supplementary Table 2. Comparisons of characteristics of participants in the overall CKB cohort, 2013-14 resurvey population, and the subpopulation in current analysis at the 2004-08 baseline survey.**

| **Characteristics** | **Overall CKB cohort** | | **2013-14 resurvey population** | | **Subpopulation in current analysis** | |
| --- | --- | --- | --- | --- | --- | --- |
|  | **Men**  **(n =** **210,205)** | **Women**  **(n =** **302,510)** | **Men**  **(n =** **9,669)** | **Women**  **(n = 15,570)** | **Men**  **(n = 3,103)** | **Women**  **(n = 5,056)** |
| Age, years | 52.9 ± 10.9 | 51.5 ± 10.5 | 52.4 ± 10.4 | 51.0 ± 10.0 | 51.2 ± 10.1 | 49.6 ± 9.7 |
| Urban residence (%) | 44.1 | 44.1 | 43.3 | 43.3 | 47.2 | 47.2 |
| No formal schooling (%) | 8.4 | 26.0 | 7.7 | 26.2 | 7.2 | 25.0 |
| High household income ^a^, % | 20.3 | 16.4 | 18.7 | 13.0 | 19.6 | 13.5 |
| Smoking, % |  |  |  |  |  |  |
| Never or occasional | 25.8 | 96.7 | 25.7 | 97.2 | 26.3 | 97.7 |
| Ex-regular | 12.9 | 0.9 | 12.6 | 0.6 | 12.510.7 | 0.4 |
| Current regular | 61.3 | 2.4 | 61.7 | 2.2 | 62.9 | 2.0 |
| Alcohol drinking, % |  |  |  |  |  |  |
| Never or occasional | 58.2 | 97.0 | 57.4 | 96.7 | 58.9 | 96.9 |
| Ex-regular | 8.5 | 0.9 | 7.8 | 1.0 | 6.2 | 0.7 |
| Current regular | 33.4 | 2.1 | 34.8 | 2.3 | 34.9 | 2.5 |
| Physical activity, MET-hr/day | 22.3 ± 15.3 | 20.2 ± 12.8 | 22.9 ± 15.6 | 20.3 ± 12.9 | 23.3 ± 15.5 | 20.8 ± 12.9 |

^a^ Annual household income ≥35,000 yuan.

Values were either percentage or mean ± SD and were adjusted for age, sex and area where appropriate. Percentages may not sum to 100 because of rounding.

**Supplementary Table 3. Median concentrations [Q1;Q3] of erythrocyte PUFAs of study participants by sex and residence (rural/urban).**

| **PUFAs** | **Males** | | **Females** | |
| --- | --- | --- | --- | --- |
|  | **Rural**  **(n = 1,672)** | **Urban**  **(n = 1,431)** | **Rural**  **(n = 2,633)** | **Urban**  **(n = 2,423)** |
| **Total n-6 PUFAs, %** | 30.4 [28.4;32.8] ^a^ | 30.6 [28.5;32.2] ^a^ | 30.2 [28.2;32.3] ^a^ | 30.6 [28.4;32.5] ^a^ |
| 18:2n-6 (linoleic acid) | 11.3 [10.2;12.7] ^a^ | 12.1 [10.9;13.4] ^b^ | 11.2 [10.1;12.7] ^a^ | 12.0 [10.8;13.3] ^b^ |
| 18:3n-6 (γ-linolenic acid) | 0.053 [0.036;0.082] ^a^ | 0.042 [0.026;0.064] ^b^ | 0.067 [0.044;0.104] ^c^ | 0.049 [0.03;0.079] ^d^ |
| 20:2n-6 (eicosadienoic acid) | 0.41 [0.37;0.46] ^a^ | 0.39 [0.35;0.43] ^b^ | 0.41 [0.36;0.46] ^a^ | 0.38 [0.34;0.42] ^c^ |
| 20:3n-6 (dihomo-γ-linolenic acid) | 1.30 [1.11;1.53] ^a^ | 1.24 [1.06;1.44] ^b^ | 1.34 [1.16;1.57] ^c^ | 1.25 [1.07;1.47] ^b^ |
| 20:4n-6 (arachidonic acid) | 13.4 [12.1;14.5] ^a^ | 13.0 [11.8;13.9] ^b^ | 13.5 [12.2;14.5] ^a^ | 13.2 [12.1;14.2] ^c^ |
| 22:2n-6 (docosadienoic acid) | 0.081 [0.066;0.101] ^a^ | 0.076 [0.061;0.095] ^b^ | 0.085 [0.069;0.107] ^c^ | 0.079 [0.062;0.099] ^d^ |
| 22:4n-6 (docosatetraenoic acid) | 2.91 [2.32;3.54] ^a^ | 2.67 [2.12;3.16] ^b^ | 2.72 [2.15;3.25] ^c^ | 2.56 [2.05;3.04] ^d^ |
| 22:5n-6 (docosapentaenoic acid) | 0.59 [0.42;0.80] ^a^ | 0.56 [0.45;0.70] ^b^ | 0.55 [0.38;0.73] ^b^ | 0.54 [0.43;0.70] ^b^ |
| **Total n-3 PUFAs; %** | 5.71 [4.98;6.61] ^a^ | 7.44 [6.14;8.69] ^b^ | 6.20 [5.44;7.14] ^c^ | 7.76 [6.50;9.08] ^d^ |
| 18:3n-3 (α-linolenic acid) | 0.20 [0.14;0.30] ^a^ | 0.15 [0.10;0.22] ^b^ | 0.24 [0.16;0.38] ^c^ | 0.15 [0.10;0.23] ^b^ |
| 20:3n-3 (eicosatrienoic acid) | 0.054 [0.039;0.081] ^a^ | 0.041 [0.03;0.055] ^b^ | 0.058 [0.041;0.089] ^c^ | 0.040 [0.029;0.053] ^b^ |
| 20:5n-3 (eicosapentaenoic acid) | 0.32 [0.21;0.49] ^a^ | 0.46 [0.30;0.69] ^b^ | 0.40 [0.27;0.60] ^c^ | 0.49 [0.32;0.72] ^d^ |
| 22:5n-3 (docosapentaenoic acid) | 1.77 [1.48;2.05] ^a^ | 1.75 [1.48;2.02] ^a^ | 1.84 [1.57;2.16] ^b^ | 1.71 [1.44;1.96] ^c^ |
| 22:6n-3 (docosahexaenoic acid) | 3.21 [2.60;3.90] ^a^ | 4.87 [3.92;5.88] ^b^ | 3.42 [2.80;4.18] ^c^ | 5.24 [4.27;6.31] ^d^ |
| **Fatty acids ratio** |  |  |  |  |
| 18:3n-6/18:2n-6 | 0.0046 [0.0031;0.0073] ^a^ | 0.0035 [0.0021;0.0053] ^b^ | 0.0059 [0.0038;0.0092] ^c^ | 0.0040 [0.0025;0.0066] ^d^ |
| 20:3n-6/18:2n-6 | 0.115 [0.095;0.136] ^a^ | 0.103 [0.085;0.122] ^b^ | 0.120 [0.099;0.142] ^c^ | 0.104 [0.087;0.125] ^b^ |
| 20:4n-6/20:3n-6 | 9.7 [8.0;11.7] ^a^ | 10.2 [8.4;12.0] ^b^ | 9.7 [8.0;11.6] ^a^ | 10.2 [8.5;12.1] ^b^ |
| Total n-6/total n-3 | 5.41 [4.50;6.37] ^a^ | 4.15 [3.41;5.16] ^b^ | 4.90 [4.06;5.83] ^c^ | 3.99 [3.26;4.87] ^d^ |

^a-d^ For each PUFA, values with different letters are significantly different from each other (*P* < 0.05). Abbreviations: PUFA, polyunsaturated fatty acid.

**Supplementary Table 4. Spearman correlations of different PUFAs.**

|  | 18:3n-6 | 20:2n-6 | 20:3n-6 | 20:4n-6 | 22:2n-6 | 22:4n-6 | 22:5n-6 | Total n-6 | 18:3n-3 | 20:3n-3 | 20:5n-3 | 22:5n-3 | 22:6n-3 | Total n-3 |
| --- | --- | --- | --- | --- | --- | --- | --- | --- | --- | --- | --- | --- | --- | --- |
| 18:2n-6 | 0.01 | 0.35*** | 0.17*** | -0.14*** | 0.16*** | -0.02 | 0.02 | 0.56*** | 0.23*** | 0 | -0.16*** | -0.13*** | -0.14*** | -0.16*** |
| 18:3n-6 |  | 0.10*** | 0.20*** | 0.01 | 0.09*** | 0.03 | 0.02 | 0.08*** | 0.22*** | 0.10*** | 0.05* | 0.07*** | -0.17*** | -0.07*** |
| 20:2n-6 |  |  | 0.42*** | 0.01 | 0.52*** | 0.20*** | 0.13*** | 0.36*** | 0.24*** | 0.21*** | -0.16*** | 0.04 | -0.20*** | -0.15*** |
| 20:3n-6 |  |  |  | 0.07*** | 0.23*** | 0.29*** | 0.30*** | 0.32*** | 0.09*** | 0.04 | -0.13*** | 0.14*** | -0.06** | -0.04 |
| 20:4n-6 |  |  |  |  | 0.04 | 0.69*** | 0.61*** | 0.60*** | -0.22*** | -0.14*** | -0.17*** | 0.15*** | 0.17*** | 0.05* |
| 22:2n-6 |  |  |  |  |  | 0.27*** | 0.16*** | 0.26*** | 0.18*** | 0.27*** | -0.10*** | 0.10*** | -0.10*** | -0.05* |
| 22:4n-6 |  |  |  |  |  |  | 0.73*** | 0.66*** | -0.25*** | -0.21*** | -0.38*** | 0.05* | 0.02 | -0.13*** |
| 22:5n-6 |  |  |  |  |  |  |  | 0.60*** | -0.26*** | -0.17*** | -0.40*** | -0.10*** | 0.10*** | -0.11*** |
| Total n-6 |  |  |  |  |  |  |  |  | -0.05* | -0.14*** | -0.39*** | -0.06** | -0.06*** | -0.20*** |
| 18:3n-3 |  |  |  |  |  |  |  |  |  | 0.51*** | 0.27*** | 0.34*** | -0.15*** | 0.12*** |
| 20:3n-3 |  |  |  |  |  |  |  |  |  |  | 0.24*** | 0.32*** | -0.07*** | 0.15*** |
| 20:5n-3 |  |  |  |  |  |  |  |  |  |  |  | 0.59*** | 0.44*** | 0.72*** |
| 22:5n-3 |  |  |  |  |  |  |  |  |  |  |  |  | 0.33*** | 0.65*** |
| 22:6n-3 |  |  |  |  |  |  |  |  |  |  |  |  |  | 0.86*** |

Spearman correlation coefficients were adjusted for age, sex, area and residence.

* *P* <0.05, ** *P*<0.001, *** *P*<0.0001.

**Supplementary Table 5. Spearman correlations of PUFAs with cardio-metabolic traits and daily dietary intake.**

|  | 18:2n-6 | 18:3n-6 | 20:2n-6 | 20:3n-6 | 20:4n-6 | 22:2n-6 | 22:4n-6 | 22:5n-6 | Total  n-6 | 18:3n-3 | 20:3n-3 | 20:5n-3 | 22:5n-3 | 22:6n-3 | Total  n-3 |
| --- | --- | --- | --- | --- | --- | --- | --- | --- | --- | --- | --- | --- | --- | --- | --- |
| BMI | 0 | 0.13*** | -0.07*** | 0.12*** | -0.06*** | -0.04** | 0.03* | -0.02 | -0.02 | -0.02 | -0.12*** | 0.02* | 0.01 | -0.01 | 0 |
| SBP | 0.03 | 0.07*** | -0.02 | 0.05*** | -0.04* | -0.01 | 0 | -0.03* | 0 | 0.06*** | -0.01 | 0.03* | 0.07*** | 0.01 | 0.03* |
| LDL-C | 0.09*** | -0.06*** | -0.06*** | 0.03* | 0.22*** | -0.01 | 0.19*** | 0.20*** | 0.23*** | -0.07*** | -0.05*** | -0.12*** | -0.01 | 0.04** | -0.05*** |
| HDL-C | -0.04* | -0.07*** | 0 | -0.08*** | 0.20*** | 0.03* | 0.14*** | 0.15*** | 0.13*** | -0.14*** | -0.01 | -0.10*** | -0.06*** | 0 | -0.09*** |
| Triglycerides | 0 | 0.26*** | -0.05*** | 0.12*** | -0.21*** | -0.13*** | -0.09*** | -0.06*** | -0.13*** | 0.11*** | -0.11*** | 0 | -0.06*** | -0.05*** | -0.03* |
| Total cholesterol | 0.06*** | 0.02 | -0.07*** | 0.02 | 0.17*** | -0.05*** | 0.17*** | 0.19*** | 0.18*** | -0.06*** | -0.08*** | -0.13*** | -0.06*** | 0.01 | -0.09*** |
| Daily dietary intake |  |  |  |  |  |  |  |  |  |  |  |  |  |  |  |
| Refined grain | -0.07*** | 0.04** | -0.04*** | -0.04** | -0.12*** | -0.03* | -0.18*** | -0.21*** | -0.18*** | 0.17*** | 0.16*** | 0.16*** | 0.13*** | 0.02 | 0.10*** |
| Coarse grain | 0.09*** | 0.10*** | 0.14*** | 0.15*** | 0.17*** | 0.16*** | 0.26*** | 0.27*** | 0.28*** | -0.12*** | -0.16*** | -0.19*** | -0.08*** | -0.04** | -0.10*** |
| Red meat | -0.03* | -0.11*** | -0.13*** | -0.05*** | 0.12*** | -0.14*** | 0.09*** | 0.10*** | 0.04** | -0.07*** | -0.01 | -0.09*** | -0.08*** | 0.01 | -0.08*** |
| Poultry | -0.03* | -0.05*** | -0.10*** | -0.04** | -0.02* | -0.10*** | -0.04** | -0.03* | -0.07*** | 0.02* | 0.05*** | 0.05*** | 0.01 | 0 | 0.01 |
| Fish | -0.06*** | -0.11*** | -0.17*** | -0.18*** | -0.10*** | -0.16*** | -0.19*** | -0.15*** | -0.19*** | -0.09*** | -0.03* | 0.18*** | 0 | 0.21*** | 0.17*** |
| Eggs | -0.04** | 0.05*** | 0.04* | -0.02 | 0.03* | 0.02 | 0.03* | 0.13*** | 0.01 | -0.06*** | -0.08*** | -0.08*** | -0.13*** | -0.03* | -0.06*** |
| Fresh vegetables | 0.04** | 0.06*** | 0.09*** | 0.07*** | 0.02* | 0.05*** | 0.04*** | 0.03* | 0.05*** | 0.03* | 0 | -0.03* | -0.02 | -0.08*** | -0.06*** |
| Soya | 0.02* | 0.03* | 0.07*** | 0.01 | -0.04*** | 0.07*** | 0 | -0.03* | 0 | 0.02 | -0.01 | -0.02* | -0.03* | -0.05*** | -0.04* |
| Fresh fruits | -0.04** | -0.01 | 0.02 | 0.04** | -0.04** | -0.01 | -0.01 | -0.01 | -0.05*** | -0.07*** | -0.07*** | -0.02* | -0.07*** | 0 | -0.02* |
| Milk | 0 | -0.01 | -0.01 | 0.02 | 0.06*** | 0.02 | 0.08*** | 0.11*** | 0.06*** | -0.04** | -0.04** | -0.08*** | -0.06*** | -0.02 | -0.05*** |

Spearman correlation coefficients were adjusted for age, sex, area, residence, education, smoking, alcohol drinking, family history of cardiovascular diseases, and physical activity. * *P* <0.05, ** *P*<0.001, *** *P*<0.0001.

Abbreviations: BMI: body mass index; HDL-C: high-density lipoprotein cholesterol; LDL-C: low-density lipoprotein cholesterol; SBP: systolic blood pressure.

**Supplementary Table 6. Adjusted hazard ratios (95% CI) for stroke events associated with n-6 PUFA quintiles in sensitivity analysis.**

|  | **Q1** | **Q2** | **Q3** | **Q4** | **Q5** | ***P_trend_*** |
| --- | --- | --- | --- | --- | --- | --- |
| **Total stroke** | | | | | | |
| 18:2n-6 | 90/1632 | 80/1632 | 74/1631 | 82/1632 | 86/1632 |  |
| Model 1 | 1.00 (0.79,1.26) | 0.95 (0.76,1.19) | 0.81 (0.65,1.02) | 0.90 (0.72,1.12) | 0.89 (0.71,1.11) | 0.42 |
| Model 2 | 1.00 (0.79,1.26) | 0.94 (0.75,1.18) | 0.82 (0.65,1.03) | 0.89 (0.71,1.11) | 0.87 (0.69,1.10) | 0.37 |
| Model 3 | 1.00 (0.77,1.29) | 0.92 (0.73,1.15) | 0.79 (0.63,0.99) | 0.84 (0.68,1.05) | 0.80 (0.62,1.04) | 0.19 |
| 18:3n-6 | 63/1631 | 81/1632 | 90/1630 | 88/1634 | 90/1632 |  |
| Model 1 | 1.00 (0.77,1.31) | 1.30 (1.04,1.62) | 1.40 (1.13,1.72) | 1.34 (1.08,1.65) | 1.28 (1.03,1.59) | 0.27 |
| Model 2 | 1.00 (0.76,1.31) | 1.31 (1.05,1.63) | 1.40 (1.13,1.73) | 1.37 (1.11,1.69) | 1.30 (1.05,1.62) | 0.22 |
| Model 3 | 1.00 (0.76,1.31) | 1.31 (1.04,1.63) | 1.40 (1.13,1.72) | 1.36 (1.10,1.68) | 1.28 (1.03,1.60) | 0.26 |
| 20:2n-6 | 80/1632 | 73/1631 | 77/1632 | 90/1632 | 92/1632 |  |
| Model 1 | 1.00 (0.78,1.28) | 0.92 (0.72,1.16) | 0.90 (0.72,1.13) | 1.05 (0.85,1.29) | 1.06 (0.85,1.32) | 0.45 |
| Model 2 | 1.00 (0.78,1.29) | 0.90 (0.71,1.14) | 0.90 (0.72,1.12) | 1.04 (0.84,1.29) | 1.04 (0.84,1.30) | 0.48 |
| Model 3 | 1.00 (0.76,1.31) | 0.89 (0.70,1.13) | 0.88 (0.70,1.10) | 1.02 (0.83,1.26) | 1.00 (0.80,1.27) | 0.61 |
| 20:3n-6 | 67/1632 | 67/1632 | 77/1631 | 92/1632 | 109/1632 |  |
| Model 1 | 1.00 (0.77,1.29) | 0.94 (0.74,1.20) | 1.02 (0.82,1.29) | 1.20 (0.98,1.48) | 1.43 (1.17,1.76) | 0.005 |
| Model 2 | 1.00 (0.77,1.30) | 0.96 (0.75,1.22) | 1.02 (0.82,1.29) | 1.22 (0.99,1.50) | 1.43 (1.17,1.76) | 0.006 |
| Model 3 | 1.00 (0.76,1.32) | 0.97 (0.76,1.24) | 1.04 (0.83,1.30) | 1.24 (1.01,1.52) | 1.46 (1.18,1.79) | 0.005 |
| 20:4n-6 | 87/1632 | 78/1632 | 86/1631 | 63/1632 | 98/1632 |  |
| Model 1 | 1.00 (0.79,1.27) | 0.92 (0.73,1.16) | 1.06 (0.86,1.31) | 0.79 (0.62,1.02) | 1.03 (0.82,1.30) | 0.88 |
| Model 2 | 1.00 (0.78,1.28) | 0.90 (0.71,1.13) | 1.02 (0.82,1.27) | 0.77 (0.60,0.99) | 1.02 (0.81,1.28) | 0.82 |
| Model 3 | 1.00 (0.75,1.33) | 0.88 (0.70,1.12) | 1.02 (0.82,1.26) | 0.77 (0.60,0.99) | 1.02 (0.80,1.30) | 0.90 |
| 22:2n-6 | 94/1632 | 75/1631 | 73/1630 | 82/1634 | 88/1632 |  |
| Model 1 | 1.00 (0.79,1.26) | 0.86 (0.69,1.08) | 0.84 (0.67,1.06) | 0.83 (0.66,1.04) | 0.83 (0.66,1.03) | 0.26 |
| Model 2 | 1.00 (0.79,1.26) | 0.85 (0.68,1.07) | 0.83 (0.66,1.05) | 0.83 (0.66,1.04) | 0.83 (0.66,1.04) | 0.28 |
| Model 3 | 1.00 (0.79,1.27) | 0.85 (0.68,1.07) | 0.83 (0.66,1.04) | 0.82 (0.65,1.03) | 0.83 (0.66,1.03) | 0.27 |
| 22:4n-6 | 84/1632 | 90/1632 | 76/1631 | 66/1632 | 96/1632 |  |
| Model 1 | 1.00 (0.78,1.29) | 1.26 (1.02,1.56) | 1.06 (0.84,1.33) | 0.94 (0.74,1.21) | 0.98 (0.74,1.31) | 0.36 |
| Model 2 | 1.00 (0.77,1.29) | 1.24 (1.00,1.54) | 1.03 (0.82,1.30) | 0.91 (0.71,1.16) | 0.96 (0.72,1.28) | 0.26 |
| Model 3 | 1.00 (0.74,1.34) | 1.24 (1.00,1.54) | 1.05 (0.83,1.32) | 0.93 (0.72,1.20) | 0.99 (0.73,1.33) | 0.33 |
| 22:5n-6 | 89/1632 | 79/1632 | 68/1631 | 70/1632 | 106/1632 |  |
| Model 1 | 1.00 (0.78,1.28) | 0.95 (0.75,1.20) | 0.88 (0.69,1.13) | 0.90 (0.71,1.15) | 0.98 (0.73,1.31) | 0.75 |
| Model 2 | 1.00 (0.77,1.29) | 0.96 (0.76,1.21) | 0.88 (0.69,1.13) | 0.89 (0.70,1.14) | 0.98 (0.73,1.31) | 0.71 |
| Model 3 | 1.00 (0.75,1.34) | 0.96 (0.76,1.22) | 0.89 (0.69,1.13) | 0.91 (0.71,1.16) | 1.00 (0.74,1.36) | 0.86 |
| Total n-6 | 88/1632 | 77/1632 | 73/1631 | 72/1632 | 102/1632 |  |
| Model 1 | 1.00 (0.78,1.29) | 0.90 (0.71,1.14) | 0.93 (0.73,1.18) | 0.79 (0.62,1.00) | 1.00 (0.77,1.30) | 0.72 |
| Model 2 | 1.00 (0.78,1.29) | 0.87 (0.69,1.11) | 0.91 (0.72,1.15) | 0.77 (0.60,0.98) | 0.98 (0.76,1.28) | 0.65 |
| Model 3 | 1.00 (0.66,1.51) | 0.82 (0.62,1.06) | 0.84 (0.67,1.05) | 0.70 (0.53,0.91) | 0.85 (0.59,1.23) | 0.38 |
| **Ischemic stroke** | | | | | | |
| 18:2n-6 | 74/1632 | 73/1632 | 63/1631 | 69/1632 | 63/1632 |  |
| Model 1 | 1.00 (0.78,1.29) | 1.02 (0.81,1.29) | 0.81 (0.63,1.04) | 0.85 (0.67,1.08) | 0.71 (0.54,0.92) | 0.032 |
| Model 2 | 1.00 (0.78,1.29) | 1.01 (0.80,1.28) | 0.81 (0.63,1.04) | 0.83 (0.65,1.06) | 0.68 (0.52,0.89) | 0.019 |
| Model 3 | 1.00 (0.75,1.32) | 0.98 (0.77,1.24) | 0.77 (0.60,0.98) | 0.78 (0.61,0.99) | 0.60 (0.45,0.81) | 0.006 |
| 18:3n-6 | 55/1631 | 63/1632 | 75/1630 | 75/1634 | 74/1632 |  |
| Model 1 | 1.00 (0.75,1.33) | 1.15 (0.90,1.49) | 1.35 (1.08,1.70) | 1.32 (1.05,1.66) | 1.27 (1.00,1.61) | 0.18 |
| Model 2 | 1.00 (0.75,1.34) | 1.16 (0.90,1.50) | 1.34 (1.07,1.69) | 1.35 (1.07,1.70) | 1.28 (1.01,1.63) | 0.15 |
| Model 3 | 1.00 (0.75,1.34) | 1.17 (0.90,1.50) | 1.35 (1.07,1.69) | 1.34 (1.07,1.69) | 1.27 (1.00,1.61) | 0.18 |
| 20:2n-6 | 67/1632 | 62/1631 | 65/1632 | 78/1632 | 70/1632 |  |
| Model 1 | 1.00 (0.76,1.32) | 0.95 (0.73,1.22) | 0.93 (0.73,1.19) | 1.15 (0.91,1.44) | 1.01 (0.79,1.29) | 0.56 |
| Model 2 | 1.00 (0.76,1.32) | 0.93 (0.72,1.20) | 0.92 (0.72,1.18) | 1.12 (0.89,1.41) | 0.98 (0.76,1.26) | 0.66 |
| Model 3 | 1.00 (0.74,1.35) | 0.93 (0.72,1.20) | 0.92 (0.72,1.18) | 1.12 (0.89,1.41) | 0.96 (0.74,1.25) | 0.69 |
| 20:3n-6 | 56/1632 | 50/1632 | 64/1631 | 78/1632 | 94/1632 |  |
| Model 1 | 1.00 (0.76,1.32) | 0.85 (0.64,1.13) | 1.05 (0.82,1.35) | 1.26 (1.01,1.58) | 1.56 (1.25,1.94) | <0.001 |
| Model 2 | 1.00 (0.75,1.33) | 0.86 (0.65,1.14) | 1.05 (0.82,1.34) | 1.28 (1.02,1.60) | 1.54 (1.23,1.91) | 0.001 |
| Model 3 | 1.00 (0.73,1.36) | 0.90 (0.68,1.19) | 1.09 (0.85,1.40) | 1.34 (1.07,1.68) | 1.63 (1.30,2.03) | <0.001 |
| 20:4n-6 | 68/1632 | 65/1632 | 72/1631 | 54/1632 | 83/1632 |  |
| Model 1 | 1.00 (0.77,1.30) | 0.97 (0.76,1.26) | 1.18 (0.93,1.48) | 0.88 (0.68,1.16) | 1.17 (0.91,1.51) | 0.57 |
| Model 2 | 1.00 (0.76,1.32) | 0.95 (0.74,1.23) | 1.13 (0.89,1.43) | 0.85 (0.65,1.12) | 1.15 (0.89,1.48) | 0.63 |
| Model 3 | 1.00 (0.72,1.38) | 1.00 (0.77,1.29) | 1.21 (0.96,1.53) | 0.94 (0.72,1.23) | 1.29 (0.99,1.68) | 0.30 |
| 22:2n-6 | 78/1632 | 67/1631 | 61/1630 | 66/1634 | 70/1632 |  |
| Model 1 | 1.00 (0.78,1.29) | 0.91 (0.71,1.15) | 0.83 (0.65,1.07) | 0.77 (0.59,0.99) | 0.77 (0.60,0.99) | 0.086 |
| Model 2 | 1.00 (0.78,1.29) | 0.90 (0.71,1.14) | 0.82 (0.64,1.06) | 0.77 (0.59,0.99) | 0.77 (0.60,0.99) | 0.093 |
| Model 3 | 1.00 (0.77,1.29) | 0.90 (0.71,1.15) | 0.83 (0.64,1.06) | 0.77 (0.60,1.00) | 0.78 (0.60,1.00) | 0.11 |
| 22:4n-6 | 65/1632 | 78/1632 | 64/1631 | 55/1632 | 80/1632 |  |
| Model 1 | 1.00 (0.75,1.33) | 1.45 (1.15,1.82) | 1.18 (0.92,1.52) | 1.04 (0.80,1.37) | 1.11 (0.81,1.51) | 0.63 |
| Model 2 | 1.00 (0.75,1.33) | 1.41 (1.12,1.78) | 1.14 (0.89,1.47) | 0.98 (0.75,1.29) | 1.06 (0.77,1.44) | 0.42 |
| Model 3 | 1.00 (0.72,1.39) | 1.50 (1.18,1.89) | 1.26 (0.98,1.61) | 1.11 (0.85,1.47) | 1.23 (0.89,1.71) | 0.84 |
| 22:5n-6 | 69/1632 | 67/1632 | 58/1631 | 62/1632 | 86/1632 |  |
| Model 1 | 1.00 (0.76,1.32) | 0.99 (0.77,1.27) | 0.93 (0.71,1.21) | 1.04 (0.80,1.34) | 1.09 (0.79,1.51) | 0.66 |
| Model 2 | 1.00 (0.75,1.33) | 1.01 (0.78,1.30) | 0.93 (0.71,1.21) | 1.03 (0.79,1.33) | 1.11 (0.80,1.53) | 0.67 |
| Model 3 | 1.00 (0.72,1.38) | 1.07 (0.83,1.38) | 1.01 (0.78,1.32) | 1.14 (0.88,1.48) | 1.27 (0.91,1.77) | 0.30 |
| Total n-6 | 72/1632 | 62/1632 | 66/1631 | 59/1632 | 83/1632 |  |
| Model 1 | 1.00 (0.76,1.32) | 0.89 (0.68,1.15) | 1.01 (0.79,1.30) | 0.74 (0.57,0.97) | 0.93 (0.70,1.24) | 0.45 |
| Model 2 | 1.00 (0.76,1.32) | 0.86 (0.66,1.12) | 0.98 (0.76,1.26) | 0.71 (0.54,0.93) | 0.89 (0.67,1.19) | 0.33 |
| Model 3 | 1.00 (0.64,1.57) | 0.84 (0.63,1.12) | 0.98 (0.77,1.24) | 0.71 (0.53,0.95) | 0.88 (0.59,1.33) | 0.46 |
| **Intracerebral hemorrhage** | | | | | | |
| 18:2n-6 | 12/1632 | 5/1632 | 8/1631 | 11/1632 | 17/1632 |  |
| Model 1 | 1.00 (0.52,1.94) | 0.58 (0.24,1.40) | 0.88 (0.44,1.79) | 1.49 (0.82,2.70) | 2.23 (1.35,3.66) | 0.009 |
| Model 2 | 1.00 (0.51,1.95) | 0.57 (0.23,1.37) | 0.90 (0.44,1.83) | 1.51 (0.84,2.74) | 2.49 (1.49,4.15) | 0.005 |
| Model 3 | 1.00 (0.47,2.11) | 0.59 (0.24,1.43) | 0.95 (0.47,1.92) | 1.62 (0.90,2.90) | 2.83 (1.56,5.13) | 0.004 |
| 18:3n-6 | 7/1631 | 12/1632 | 10/1630 | 11/1634 | 13/1632 |  |
| Model 1 | 1.00 (0.45,2.20) | 1.57 (0.88,2.79) | 1.16 (0.60,2.24) | 1.38 (0.76,2.50) | 1.21 (0.66,2.22) | 0.98 |
| Model 2 | 1.00 (0.45,2.22) | 1.66 (0.93,2.97) | 1.29 (0.67,2.50) | 1.40 (0.76,2.55) | 1.21 (0.66,2.24) | 0.96 |
| Model 3 | 1.00 (0.45,2.21) | 1.65 (0.92,2.95) | 1.27 (0.66,2.46) | 1.40 (0.77,2.55) | 1.19 (0.64,2.21) | 0.94 |
| 20:2n-6 | 10/1632 | 7/1631 | 7/1632 | 10/1632 | 19/1632 |  |
| Model 1 | 1.00 (0.49,2.06) | 0.74 (0.35,1.56) | 0.69 (0.33,1.45) | 0.84 (0.44,1.62) | 1.62 (0.97,2.70) | 0.15 |
| Model 2 | 1.00 (0.47,2.12) | 0.76 (0.36,1.61) | 0.73 (0.35,1.53) | 0.94 (0.49,1.82) | 1.77 (1.05,2.98) | 0.096 |
| Model 3 | 1.00 (0.45,2.21) | 0.74 (0.35,1.58) | 0.70 (0.33,1.47) | 0.91 (0.47,1.76) | 1.76 (1.01,3.07) | 0.11 |
| 20:3n-6 | 10/1632 | 12/1632 | 8/1631 | 12/1632 | 11/1632 |  |
| Model 1 | 1.00 (0.52,1.93) | 1.12 (0.61,2.04) | 0.69 (0.33,1.46) | 1.06 (0.60,1.87) | 0.96 (0.51,1.80) | 0.90 |
| Model 2 | 1.00 (0.51,1.97) | 1.06 (0.58,1.95) | 0.66 (0.31,1.40) | 1.06 (0.60,1.88) | 1.00 (0.53,1.88) | 0.99 |
| Model 3 | 1.00 (0.49,2.03) | 0.95 (0.52,1.75) | 0.59 (0.28,1.24) | 0.94 (0.53,1.66) | 0.86 (0.46,1.64) | 0.81 |
| 20:4n-6 | 15/1632 | 11/1632 | 13/1631 | 5/1632 | 9/1632 |  |
| Model 1 | 1.00 (0.55,1.80) | 0.79 (0.44,1.44) | 0.84 (0.47,1.53) | 0.43 (0.18,1.06) | 0.68 (0.33,1.42) | 0.25 |
| Model 2 | 1.00 (0.55,1.83) | 0.77 (0.42,1.41) | 0.82 (0.45,1.50) | 0.42 (0.17,1.03) | 0.63 (0.30,1.32) | 0.20 |
| Model 3 | 1.00 (0.51,1.94) | 0.59 (0.32,1.07) | 0.59 (0.32,1.07) | 0.28 (0.12,0.69) | 0.38 (0.18,0.82) | 0.029 |
| 22:2n-6 | 13/1632 | 4/1631 | 9/1630 | 12/1634 | 15/1632 |  |
| Model 1 | 1.00 (0.52,1.94) | 0.38 (0.14,1.00) | 0.86 (0.45,1.66) | 1.17 (0.66,2.08) | 1.42 (0.82,2.44) | 0.14 |
| Model 2 | 1.00 (0.51,1.97) | 0.37 (0.14,0.98) | 0.87 (0.45,1.68) | 1.17 (0.66,2.08) | 1.43 (0.82,2.49) | 0.14 |
| Model 3 | 1.00 (0.50,1.98) | 0.35 (0.13,0.95) | 0.83 (0.43,1.60) | 1.13 (0.63,2.00) | 1.37 (0.78,2.38) | 0.16 |
| 22:4n-6 | 17/1632 | 11/1632 | 9/1631 | 7/1632 | 9/1632 |  |
| Model 1 | 1.00 (0.54,1.84) | 0.66 (0.36,1.22) | 0.41 (0.20,0.84) | 0.46 (0.21,0.99) | 0.46 (0.20,1.09) | 0.064 |
| Model 2 | 1.00 (0.53,1.88) | 0.65 (0.35,1.21) | 0.38 (0.18,0.79) | 0.48 (0.22,1.04) | 0.50 (0.21,1.19) | 0.098 |
| Model 3 | 1.00 (0.49,2.06) | 0.46 (0.25,0.85) | 0.22 (0.11,0.46) | 0.26 (0.12,0.57) | 0.23 (0.10,0.58) | 0.004 |
| 22:5n-6 | 19/1632 | 10/1632 | 6/1631 | 5/1632 | 13/1632 |  |
| Model 1 | 1.00 (0.53,1.87) | 0.69 (0.37,1.29) | 0.43 (0.19,0.97) | 0.33 (0.13,0.80) | 0.53 (0.23,1.23) | 0.067 |
| Model 2 | 1.00 (0.53,1.89) | 0.63 (0.34,1.17) | 0.38 (0.17,0.86) | 0.27 (0.11,0.66) | 0.43 (0.18,1.02) | 0.026 |
| Model 3 | 1.00 (0.50,2.00) | 0.48 (0.26,0.89) | 0.27 (0.12,0.61) | 0.18 (0.07,0.45) | 0.26 (0.11,0.64) | 0.002 |
| Total n-6 | 14/1632 | 12/1632 | 5/1631 | 11/1632 | 11/1632 |  |
| Model 1 | 1.00 (0.52,1.91) | 0.77 (0.40,1.46) | 0.49 (0.20,1.17) | 1.26 (0.68,2.34) | 1.45 (0.69,3.05) | 0.28 |
| Model 2 | 1.00 (0.51,1.94) | 0.78 (0.41,1.49) | 0.51 (0.21,1.23) | 1.44 (0.77,2.69) | 1.70 (0.80,3.62) | 0.14 |
| Model 3 | 1.00 (0.32,3.09) | 0.59 (0.28,1.23) | 0.34 (0.14,0.80) | 0.83 (0.41,1.68) | 0.82 (0.28,2.39) | 0.81 |

Model 1, on top of basic model, further adjusted for SBP, BMI and LDL-C;

Model 2, on top of model 1, further adjusted for fasting hours, total n-3 PUFA, and daily dietary intake of refined grain, coarse grain, red meat, poultry, fish, eggs, fresh vegetables, soya, fresh fruits and milk;

Model 3, on top of model 2, further adjusted for saturated fatty acids and monounsaturated fatty acids.

Cox regression was used to estimate hazard ratios and 95% confidence intervals. For analyses involving more than two exposure categories, the floating absolute-risk method was applied to provide 95% CI for each category.

Abbreviations: CI, confidence interval; SD, standard deviation; PUFA, polyunsaturated fatty acid.

**Supplementary Table 7. Adjusted hazard ratios (95% CI) for stroke events associated with n-3 PUFA quintiles in sensitivity analysis.**

|  | **Q1** | **Q2** | **Q3** | **Q4** | **Q5** | ***P_trend_*** |
| --- | --- | --- | --- | --- | --- | --- |
| **Total stroke** | | | | | | |
| 18:3n-3 | 87/1632 | 84/1632 | 76/1631 | 64/1632 | 101/1632 |  |
| Model 1 | 1.00 (0.77,1.29) | 0.90 (0.72,1.13) | 0.84 (0.67,1.05) | 0.66 (0.50,0.86) | 1.05 (0.81,1.37) | 0.58 |
| Model 2 | 1.00 (0.77,1.29) | 0.88 (0.70,1.11) | 0.83 (0.66,1.04) | 0.65 (0.50,0.84) | 1.04 (0.80,1.36) | 0.56 |
| Model 3 | 1.00 (0.77,1.30) | 0.87 (0.69,1.10) | 0.82 (0.65,1.02) | 0.62 (0.48,0.81) | 0.96 (0.71,1.28) | 0.24 |
| 20:3n-3 | 79/1631 | 81/1633 | 82/1630 | 79/1633 | 91/1632 |  |
| Model 1 | 1.00 (0.78,1.28) | 0.96 (0.77,1.21) | 1.03 (0.83,1.29) | 0.95 (0.76,1.20) | 1.17 (0.90,1.51) | 0.50 |
| Model 2 | 1.00 (0.78,1.28) | 0.96 (0.76,1.20) | 1.05 (0.84,1.30) | 0.96 (0.76,1.21) | 1.17 (0.91,1.52) | 0.46 |
| Model 3 | 1.00 (0.78,1.29) | 0.95 (0.75,1.19) | 1.03 (0.83,1.28) | 0.93 (0.74,1.17) | 1.11 (0.84,1.45) | 0.70 |
| 20:5n-3 | 89/1632 | 63/1632 | 78/1631 | 93/1632 | 89/1632 |  |
| Model 1 | 1.00 (0.77,1.29) | 0.73 (0.56,0.95) | 0.98 (0.78,1.23) | 1.24 (1.00,1.54) | 1.03 (0.81,1.31) | 0.11 |
| Model 2 | 1.00 (0.77,1.30) | 0.73 (0.56,0.95) | 0.98 (0.78,1.23) | 1.26 (1.02,1.56) | 1.07 (0.84,1.36) | 0.070 |
| Model 3 | 1.00 (0.75,1.34) | 0.73 (0.56,0.95) | 0.97 (0.77,1.21) | 1.24 (1.00,1.54) | 1.06 (0.79,1.42) | 0.071 |
| 22:5n-3 | 74/1632 | 73/1632 | 91/1631 | 67/1632 | 107/1632 |  |
| Model 1 | 1.00 (0.78,1.28) | 0.94 (0.74,1.19) | 1.30 (1.06,1.60) | 0.87 (0.68,1.11) | 1.25 (1.01,1.54) | 0.27 |
| Model 2 | 1.00 (0.77,1.29) | 0.93 (0.74,1.19) | 1.30 (1.05,1.60) | 0.88 (0.69,1.12) | 1.27 (1.03,1.58) | 0.22 |
| Model 3 | 1.00 (0.74,1.35) | 0.94 (0.73,1.19) | 1.31 (1.07,1.61) | 0.89 (0.69,1.14) | 1.29 (1.01,1.64) | 0.27 |
| 22:6n-3 | 85/1632 | 88/1632 | 76/1631 | 83/1632 | 80/1632 |  |
| Model 1 | 1.00 (0.77,1.30) | 0.94 (0.75,1.19) | 0.90 (0.71,1.14) | 1.15 (0.90,1.46) | 0.95 (0.71,1.27) | 0.72 |
| Model 2 | 1.00 (0.76,1.31) | 0.94 (0.74,1.19) | 0.91 (0.72,1.14) | 1.15 (0.90,1.46) | 0.99 (0.74,1.33) | 0.60 |
| Model 3 | 1.00 (0.72,1.39) | 0.93 (0.73,1.19) | 0.88 (0.70,1.10) | 1.09 (0.83,1.42) | 0.89 (0.58,1.36) | 0.91 |
| Total n-3 | 86/1632 | 76/1632 | 71/1631 | 98/1632 | 81/1632 |  |
| Model 1 | 1.00 (0.79,1.26) | 0.91 (0.72,1.17) | 0.88 (0.69,1.11) | 1.30 (1.04,1.61) | 1.03 (0.79,1.35) | 0.23 |
| Model 2 | 1.00 (0.79,1.27) | 0.92 (0.72,1.17) | 0.88 (0.69,1.12) | 1.31 (1.05,1.63) | 1.08 (0.82,1.42) | 0.16 |
| Model 3 | 1.00 (0.70,1.43) | 0.93 (0.71,1.21) | 0.90 (0.72,1.13) | 1.37 (1.04,1.82) | 1.19 (0.73,1.94) | 0.12 |
| **Ischemic stroke** | | | | | | |
| 18:3n-3 | 73/1632 | 71/1632 | 67/1631 | 53/1632 | 78/1632 |  |
| Model 1 | 1.00 (0.75,1.33) | 0.93 (0.72,1.19) | 0.90 (0.71,1.14) | 0.68 (0.51,0.91) | 1.04 (0.77,1.39) | 0.57 |
| Model 2 | 1.00 (0.75,1.33) | 0.91 (0.71,1.17) | 0.90 (0.71,1.14) | 0.66 (0.50,0.89) | 1.02 (0.76,1.37) | 0.52 |
| Model 3 | 1.00 (0.75,1.33) | 0.90 (0.70,1.15) | 0.87 (0.69,1.11) | 0.62 (0.47,0.83) | 0.89 (0.65,1.23) | 0.17 |
| 20:3n-3 | 70/1631 | 68/1633 | 73/1630 | 61/1633 | 70/1632 |  |
| Model 1 | 1.00 (0.77,1.30) | 0.89 (0.70,1.15) | 1.02 (0.81,1.29) | 0.85 (0.65,1.09) | 1.06 (0.79,1.41) | 0.95 |
| Model 2 | 1.00 (0.77,1.30) | 0.88 (0.69,1.13) | 1.04 (0.82,1.31) | 0.85 (0.65,1.10) | 1.06 (0.79,1.41) | 0.92 |
| Model 3 | 1.00 (0.76,1.31) | 0.87 (0.68,1.11) | 1.02 (0.81,1.28) | 0.81 (0.63,1.05) | 0.97 (0.71,1.32) | 0.72 |
| 20:5n-3 | 71/1632 | 54/1632 | 61/1631 | 84/1632 | 72/1632 |  |
| Model 1 | 1.00 (0.75,1.33) | 0.79 (0.60,1.04) | 0.96 (0.75,1.24) | 1.40 (1.12,1.75) | 1.01 (0.78,1.31) | 0.11 |
| Model 2 | 1.00 (0.75,1.33) | 0.79 (0.59,1.04) | 0.96 (0.74,1.24) | 1.42 (1.13,1.78) | 1.06 (0.81,1.38) | 0.068 |
| Model 3 | 1.00 (0.72,1.38) | 0.78 (0.59,1.04) | 0.94 (0.73,1.21) | 1.39 (1.10,1.75) | 1.04 (0.75,1.43) | 0.069 |
| 22:5n-3 | 64/1632 | 57/1632 | 77/1631 | 57/1632 | 87/1632 |  |
| Model 1 | 1.00 (0.77,1.31) | 0.86 (0.66,1.12) | 1.28 (1.02,1.60) | 0.87 (0.66,1.13) | 1.20 (0.95,1.52) | 0.32 |
| Model 2 | 1.00 (0.76,1.32) | 0.88 (0.67,1.15) | 1.31 (1.04,1.64) | 0.89 (0.69,1.16) | 1.25 (0.99,1.58) | 0.23 |
| Model 3 | 1.00 (0.72,1.38) | 0.88 (0.67,1.15) | 1.32 (1.06,1.65) | 0.91 (0.69,1.18) | 1.27 (0.98,1.65) | 0.26 |
| 22:6n-3 | 62/1632 | 74/1632 | 63/1631 | 72/1632 | 71/1632 |  |
| Model 1 | 1.00 (0.74,1.34) | 1.04 (0.80,1.34) | 0.97 (0.75,1.25) | 1.23 (0.95,1.59) | 1.02 (0.75,1.39) | 0.57 |
| Model 2 | 1.00 (0.73,1.36) | 1.07 (0.82,1.39) | 1.02 (0.79,1.32) | 1.28 (0.99,1.66) | 1.12 (0.82,1.53) | 0.35 |
| Model 3 | 1.00 (0.68,1.47) | 1.10 (0.84,1.45) | 1.06 (0.83,1.36) | 1.35 (1.01,1.80) | 1.20 (0.76,1.91) | 0.27 |
| Total n-3 | 70/1632 | 56/1632 | 64/1631 | 84/1632 | 68/1632 |  |
| Model 1 | 1.00 (0.77,1.29) | 0.83 (0.62,1.09) | 0.96 (0.75,1.23) | 1.29 (1.02,1.63) | 0.97 (0.72,1.30) | 0.30 |
| Model 2 | 1.00 (0.77,1.31) | 0.85 (0.64,1.13) | 0.99 (0.77,1.27) | 1.33 (1.05,1.69) | 1.05 (0.78,1.41) | 0.17 |
| Model 3 | 1.00 (0.67,1.49) | 0.86 (0.63,1.16) | 1.00 (0.79,1.26) | 1.38 (1.02,1.86) | 1.13 (0.66,1.93) | 0.11 |
| **Intracerebral hemorrhage** | | | | | | |
| 18:3n-3 | 8/1632 | 8/1632 | 8/1631 | 9/1632 | 20/1632 |  |
| Model 1 | 1.00 (0.43,2.34) | 0.99 (0.48,2.07) | 0.93 (0.46,1.86) | 0.79 (0.37,1.70) | 1.33 (0.66,2.65) | 0.71 |
| Model 2 | 1.00 (0.43,2.33) | 0.96 (0.46,1.99) | 0.92 (0.45,1.85) | 0.79 (0.37,1.69) | 1.32 (0.65,2.66) | 0.71 |
| Model 3 | 1.00 (0.43,2.34) | 0.97 (0.47,2.01) | 0.95 (0.47,1.92) | 0.86 (0.40,1.84) | 1.61 (0.74,3.47) | 0.50 |
| 20:3n-3 | 3/1631 | 7/1633 | 8/1630 | 14/1633 | 21/1632 |  |
| Model 1 | 1.00 (0.30,3.32) | 2.76 (1.25,6.09) | 3.14 (1.53,6.46) | 4.33 (2.48,7.54) | 6.12 (3.40,11.0) | 0.006 |
| Model 2 | 1.00 (0.30,3.36) | 3.03 (1.36,6.74) | 3.41 (1.66,7.01) | 4.76 (2.72,8.34) | 6.78 (3.81,12.1) | 0.004 |
| Model 3 | 1.00 (0.30,3.37) | 3.00 (1.35,6.68) | 3.41 (1.66,7.00) | 4.96 (2.83,8.68) | 7.76 (4.31,14.0) | 0.002 |
| 20:5n-3 | 8/1632 | 9/1632 | 15/1631 | 8/1632 | 13/1632 |  |
| Model 1 | 1.00 (0.45,2.23) | 1.16 (0.57,2.37) | 2.05 (1.19,3.53) | 1.08 (0.53,2.18) | 1.66 (0.88,3.15) | 0.46 |
| Model 2 | 1.00 (0.44,2.26) | 1.08 (0.53,2.22) | 1.90 (1.10,3.29) | 0.97 (0.48,1.98) | 1.52 (0.78,2.94) | 0.58 |
| Model 3 | 1.00 (0.41,2.41) | 1.07 (0.51,2.22) | 1.90 (1.11,3.25) | 0.96 (0.47,1.97) | 1.39 (0.62,3.16) | 0.74 |
| 22:5n-3 | 5/1632 | 12/1632 | 11/1631 | 8/1632 | 17/1632 |  |
| Model 1 | 1.00 (0.41,2.46) | 2.35 (1.26,4.37) | 2.19 (1.20,3.99) | 1.35 (0.64,2.85) | 2.24 (1.24,4.05) | 0.55 |
| Model 2 | 1.00 (0.39,2.55) | 2.05 (1.09,3.84) | 1.90 (1.04,3.47) | 1.23 (0.58,2.61) | 2.02 (1.11,3.69) | 0.62 |
| Model 3 | 1.00 (0.35,2.87) | 2.06 (1.09,3.92) | 1.90 (1.06,3.44) | 1.23 (0.58,2.61) | 2.00 (1.02,3.93) | 0.76 |
| 22:6n-3 | 18/1632 | 10/1632 | 11/1631 | 8/1632 | 6/1632 |  |
| Model 1 | 1.00 (0.54,1.84) | 0.82 (0.43,1.57) | 1.19 (0.63,2.25) | 1.21 (0.55,2.67) | 0.77 (0.28,2.14) | 0.89 |
| Model 2 | 1.00 (0.53,1.88) | 0.79 (0.41,1.52) | 1.12 (0.59,2.13) | 1.10 (0.49,2.46) | 0.61 (0.21,1.73) | 0.83 |
| Model 3 | 1.00 (0.48,2.07) | 0.64 (0.33,1.22) | 0.78 (0.41,1.47) | 0.62 (0.26,1.47) | 0.21 (0.05,0.82) | 0.13 |
| Total n-3 | 10/1632 | 16/1632 | 5/1631 | 12/1632 | 10/1632 |  |
| Model 1 | 1.00 (0.51,1.97) | 1.76 (1.00,3.08) | 0.53 (0.20,1.42) | 1.97 (1.07,3.63) | 1.90 (0.90,4.04) | 0.24 |
| Model 2 | 1.00 (0.50,2.01) | 1.49 (0.85,2.64) | 0.47 (0.18,1.27) | 1.79 (0.96,3.31) | 1.64 (0.75,3.56) | 0.33 |
| Model 3 | 1.00 (0.36,2.76) | 1.55 (0.85,2.83) | 0.50 (0.19,1.30) | 1.89 (0.84,4.25) | 1.74 (0.44,6.88) | 0.58 |

Model 1, on top of basic model, further adjusted for SBP, BMI and LDL-C;

Model 2, on top of model 1, further adjusted for fasting hours, total n-6 PUFA, and daily dietary intake of refined grain, coarse grain, red meat, poultry, fish, eggs, fresh vegetables, soya, fresh fruits and milk.

Model 3, on top of model 2, further adjusted for saturated fatty acids and monounsaturated fatty acids.

Cox regression was used to estimate hazard ratios and 95% confidence intervals. For analyses involving more than two exposure categories, the floating absolute-risk method was applied to provide 95% CI for each category.

Abbreviations: CI, confidence interval; SD, standard deviation; PUFA, polyunsaturated fatty acid.

**Supplementary Table 8. Adjusted hazard ratios (95% CI) for stroke events associated with 20:5n-3 quintiles.**

|  | **Q1** | **Q2** | **Q3** | **Q4** | **Q5** | ***P*_trend_** |
| --- | --- | --- | --- | --- | --- | --- |
| **Total stroke** | | | | | | |
| Model 1 | 1.00 (0.77,1.30) | 0.75 (0.58,0.97) | 1.05 (0.84,1.32) | 1.35 (1.09,1.67) | 1.12 (0.89,1.42) | 0.029 |
| Model 2 | 1.00 (0.77-1.29) | 0.74 (0.57-0.96) | 1.02 (0.81-1.28) | 1.30 (1.05-1.60) | 1.08 (0.85-1.37) | 0.061 |
| Model 3 | 1.00 (0.77-1.29) | 0.73 (0.56-0.94) | 0.99 (0.79-1.25) | 1.27 (1.02-1.57) | 1.05 (0.83-1.33) | 0.082 |
| Model 4 | 1.00 (0.77-1.30) | 0.75 (0.58-0.97) | 1.05 (0.84-1.32) | 1.35 (1.09-1.67) | 1.12 (0.89-1.42) | 0.030 |
| Model 5 | 1.00 (0.77-1.29) | 0.73 (0.56-0.95) | 0.98 (0.78-1.23) | 1.24 (1.00-1.54) | 1.03 (0.81-1.31) | 0.11 |
| **Ischemic stroke** | | | | | | |
| Model 1 | 1.00 (0.75,1.34) | 0.81 (0.61,1.07) | 1.03 (0.80,1.33) | 1.51 (1.21,1.89) | 1.10 (0.85,1.43) | 0.036 |
| Model 2 | 1.00 (0.75-1.33) | 0.80 (0.61-1.07) | 1.00 (0.78-1.29) | 1.46 (1.17-1.83) | 1.06 (0.82-1.38) | 0.061 |
| Model 3 | 1.00 (0.75-1.33) | 0.79 (0.59-1.04) | 0.97 (0.75-1.25) | 1.42 (1.13-1.78) | 1.03 (0.79-1.34) | 0.089 |
| Model 4 | 1.00 (0.75-1.34) | 0.81 (0.61-1.07) | 1.03 (0.80-1.33) | 1.51 (1.21-1.89) | 1.10 (0.85-1.43) | 0.037 |
| Model 5 | 1.00 (0.75-1.33) | 0.79 (0.60-1.04) | 0.96 (0.75-1.24) | 1.40 (1.12-1.75) | 1.01 (0.78-1.31) | 0.11 |

Model 1 was adjusted for age, sex, study areas, education, smoking, alcohol drinking, family history of cardiovascular diseases, and physical activity;

Model 2, on top of model 1, further adjusted for SBP;

Model 3, on top of model 1, further adjusted for BMI;

Model 4, on top of model 1, further adjusted for LDL;

Model 5, on top of model 1, further adjusted for SBP, BMI and LDL.

Cox regression was used to estimate hazard ratios and 95% confidence intervals. For analyses involving more than two exposure categories, the floating absolute-risk method was applied to provide 95% CI for each category.

**Supplementary Table 9. Adjusted hazard ratios (95% CI) for stroke events associated with PUFA ratio quintiles.**

|  | **Q1** | **Q2** | **Q3** | **Q4** | **Q5** | ***P_trend_*** |
| --- | --- | --- | --- | --- | --- | --- |
| **Total stroke** | | | | | | |
| 18:3n-6/18:2n-6 | 66/1632 | 82/1632 | 91/1631 | 70/1632 | 103/1632 |  |
| Model 1 | 1.00 (0.77,1.30) | 1.27 (1.02,1.59) | 1.38 (1.12,1.69) | 1.10 (0.87,1.40) | 1.54 (1.26,1.89) | 0.051 |
| Model 2 | 1.00 (0.77,1.30) | 1.27 (1.02,1.59) | 1.34 (1.09,1.66) | 1.02 (0.81,1.30) | 1.44 (1.17,1.77) | 0.17 |
| Model 3 | 1.00 (0.77,1.30) | 1.29 (1.03,1.61) | 1.36 (1.10,1.68) | 1.04 (0.82,1.32) | 1.47 (1.20,1.80) | 0.14 |
| 20:3n-6/18:2n-6 | 64/1632 | 76/1632 | 72/1631 | 101/1632 | 99/1632 |  |
| Model 1 | 1.00 (0.77,1.29) | 1.17 (0.93,1.47) | 1.14 (0.91,1.44) | 1.61 (1.31,1.97) | 1.50 (1.21,1.86) | 0.002 |
| Model 2 | 1.00 (0.77,1.29) | 1.14 (0.90,1.43) | 1.09 (0.87,1.38) | 1.52 (1.24,1.86) | 1.42 (1.15,1.76) | 0.007 |
| Model 3 | 1.00 (0.77,1.30) | 1.17 (0.92,1.47) | 1.11 (0.88,1.40) | 1.55 (1.27,1.9) | 1.45 (1.18,1.80) | 0.005 |
| 20:4n-6/20:3n-6 | 87/1632 | 105/1632 | 77/1631 | 80/1632 | 63/1632 |  |
| Model 1 | 1.00 (0.80,1.25) | 1.14 (0.94,1.38) | 0.84 (0.67,1.05) | 0.85 (0.68,1.06) | 0.73 (0.57,0.94) | 0.010 |
| Model 2 | 1.00 (0.80,1.25) | 1.21 (0.99,1.47) | 0.86 (0.69,1.08) | 0.92 (0.73,1.15) | 0.83 (0.64,1.07) | 0.067 |
| Model 3 | 1.00 (0.80,1.25) | 1.23 (1.01,1.49) | 0.88 (0.70,1.10) | 0.93 (0.74,1.16) | 0.84 (0.65,1.08) | 0.071 |
| Total n-6/n-3 | 80/1632 | 92/1632 | 85/1631 | 69/1632 | 86/1632 |  |
| Model 1 | 1.00 (0.76,1.31) | 1.22 (0.98,1.52) | 0.97 (0.78,1.20) | 0.80 (0.62,1.03) | 0.88 (0.69,1.12) | 0.064 |
| Model 2 | 1.00 (0.76,1.31) | 1.23 (0.99,1.54) | 0.99 (0.79,1.23) | 0.81 (0.63,1.04) | 0.94 (0.74,1.20) | 0.13 |
| Model 3 | 1.00 (0.76,1.31) | 1.20 (0.96,1.49) | 0.96 (0.77,1.19) | 0.77 (0.60,1.00) | 0.90 (0.71,1.15) | 0.078 |
| **Ischemic stroke** | | | | | | |
| 18:3n-6/18:2n-6 | 55/1632 | 64/1632 | 77/1631 | 60/1632 | 86/1632 |  |
| Model 1 | 1.00 (0.75,1.33) | 1.21 (0.95,1.56) | 1.44 (1.15,1.81) | 1.20 (0.93,1.56) | 1.66 (1.33,2.07) | 0.012 |
| Model 2 | 1.00 (0.75,1.33) | 1.21 (0.94,1.55) | 1.40 (1.12,1.76) | 1.11 (0.86,1.44) | 1.56 (1.25,1.94) | 0.045 |
| Model 3 | 1.00 (0.75,1.33) | 1.22 (0.95,1.57) | 1.41 (1.12,1.77) | 1.13 (0.87,1.46) | 1.59 (1.27,1.99) | 0.036 |
| 20:3n-6/18:2n-6 | 50/1632 | 62/1632 | 60/1631 | 84/1632 | 86/1632 |  |
| Model 1 | 1.00 (0.75,1.34) | 1.24 (0.96,1.60) | 1.28 (0.99,1.65) | 1.88 (1.50,2.34) | 1.84 (1.47,2.32) | <0.001 |
| Model 2 | 1.00 (0.75,1.34) | 1.21 (0.94,1.57) | 1.22 (0.95,1.57) | 1.77 (1.42,2.21) | 1.75 (1.39,2.21) | <0.001 |
| Model 3 | 1.00 (0.75,1.34) | 1.24 (0.96,1.61) | 1.24 (0.96,1.60) | 1.80 (1.44,2.25) | 1.78 (1.41,2.24) | <0.001 |
| 20:4n-6/20:3n-6 | 72/1632 | 87/1632 | 66/1631 | 65/1632 | 52/1632 |  |
| Model 1 | 1.00 (0.79,1.27) | 1.11 (0.90,1.38) | 0.86 (0.67,1.09) | 0.81 (0.63,1.04) | 0.70 (0.53,0.92) | 0.010 |
| Model 2 | 1.00 (0.78,1.28) | 1.17 (0.94,1.45) | 0.88 (0.69,1.12) | 0.87 (0.67,1.11) | 0.79 (0.60,1.05) | 0.054 |
| Model 3 | 1.00 (0.78,1.28) | 1.20 (0.97,1.49) | 0.89 (0.70,1.14) | 0.88 (0.69,1.14) | 0.80 (0.61,1.07) | 0.060 |
| Total n-6/n-3 | 65/1632 | 84/1632 | 71/1631 | 51/1632 | 71/1632 |  |
| Model 1 | 1.00 (0.74,1.34) | 1.43 (1.13,1.80) | 1.05 (0.82,1.33) | 0.77 (0.58,1.03) | 0.95 (0.72,1.24) | 0.055 |
| Model 2 | 1.00 (0.74,1.35) | 1.45 (1.15,1.82) | 1.08 (0.85,1.37) | 0.79 (0.59,1.05) | 1.01 (0.77,1.33) | 0.11 |
| Model 3 | 1.00 (0.74,1.35) | 1.38 (1.10,1.74) | 1.02 (0.80,1.29) | 0.74 (0.55,0.99) | 0.94 (0.72,1.24) | 0.056 |
| **Intracerebral hemorrhage** | | | | | | |
| 18:3n-6/18:2n-6 | 9/1632 | 12/1632 | 9/1631 | 9/1632 | 14/1632 |  |
| Model 1 | 1.00 (0.49,2.03) | 1.26 (0.71,2.24) | 0.83 (0.43,1.59) | 0.75 (0.37,1.50) | 1.09 (0.61,1.94) | 0.75 |
| Model 2 | 1.00 (0.50,2.02) | 1.31 (0.73,2.34) | 0.83 (0.43,1.61) | 0.72 (0.36,1.45) | 0.97 (0.54,1.75) | 0.52 |
| Model 3 | 1.00 (0.49,2.03) | 1.34 (0.75,2.40) | 0.88 (0.45,1.70) | 0.72 (0.36,1.45) | 0.92 (0.51,1.66) | 0.42 |
| 20:3n-6/18:2n-6 | 13/1632 | 8/1632 | 9/1631 | 14/1632 | 9/1632 |  |
| Model 1 | 1.00 (0.56,1.78) | 0.60 (0.30,1.20) | 0.63 (0.33,1.22) | 0.80 (0.45,1.41) | 0.52 (0.27,1.03) | 0.30 |
| Model 2 | 1.00 (0.56,1.79) | 0.53 (0.26,1.07) | 0.59 (0.31,1.14) | 0.76 (0.43,1.36) | 0.48 (0.24,0.94) | 0.25 |
| Model 3 | 1.00 (0.55,1.80) | 0.53 (0.26,1.07) | 0.60 (0.31,1.16) | 0.79 (0.45,1.41) | 0.51 (0.26,1.02) | 0.34 |
| 20:4n-6/20:3n-6 | 10/1632 | 16/1632 | 8/1631 | 12/1632 | 7/1632 |  |
| Model 1 | 1.00 (0.52,1.91) | 1.82 (1.11,2.98) | 0.84 (0.40,1.77) | 1.30 (0.71,2.36) | 0.90 (0.42,1.95) | 0.53 |
| Model 2 | 1.00 (0.52,1.92) | 2.11 (1.29,3.47) | 0.87 (0.41,1.83) | 1.45 (0.80,2.64) | 1.07 (0.50,2.30) | 0.67 |
| Model 3 | 1.00 (0.52,1.92) | 2.04 (1.24,3.36) | 0.83 (0.39,1.76) | 1.38 (0.75,2.51) | 1.01 (0.46,2.18) | 0.60 |
| Total n-6/n-3 | 12/1632 | 7/1632 | 10/1631 | 16/1632 | 8/1632 |  |
| Model 1 | 1.00 (0.50,2.01) | 0.48 (0.23,1.04) | 0.59 (0.30,1.13) | 1.00 (0.58,1.74) | 0.46 (0.22,0.95) | 0.60 |
| Model 2 | 1.00 (0.49,2.03) | 0.48 (0.22,1.03) | 0.56 (0.29,1.09) | 0.92 (0.53,1.62) | 0.48 (0.23,1.01) | 0.59 |
| Model 3 | 1.00 (0.49,2.06) | 0.51 (0.24,1.10) | 0.60 (0.31,1.17) | 0.98 (0.56,1.73) | 0.52 (0.25,1.09) | 0.69 |

Model 1, adjusted for age, sex, study areas, education, smoking, alcohol drinking, family history of cardiovascular diseases, and physical activity;

Model 2, on top of model 1, further adjusted for SBP, BMI and LDL-C;

Model 3, on top of model 2, further adjusted for fasting hours, and daily dietary intake of refined grain, coarse grain, red meat, poultry, fish, eggs, fresh vegetables, soya, fresh fruits and milk.

Cox regression was used to estimate hazard ratios and 95% confidence intervals. For analyses involving more than two exposure categories, the floating absolute-risk method was applied to provide 95% CI for each category.

Abbreviations: CI, confidence interval; SD, standard deviation; PUFA, polyunsaturated fatty acid.

**Supplementary Table 10. Associations of PUFAs with risks of ischemic stroke in subgroups analysis by study areas.**

| Study area | Case number | HR (95% CI) per SD | | | |
| --- | --- | --- | --- | --- | --- |
|  |  | 18:2n-6 | 20:3n-6 | 22:2n-6 | 20:5n-3 |
| Qingdao | 18 | 1.51 (0.92,2.49) | 1.39 (0.83,2.31) | 0.90 (0.52,1.56) | 1.19 (0.71,2.00) |
| Harbin | 57 | 0.97 (0.72,1.30) | 1.02 (0.78,1.33) | 0.92 (0.71,1.17) | 0.99 (0.76,1.29) |
| Haikou | 54 | 0.92 (0.70,1.22) | 1.33 (1.03,1.70) | 0.87 (0.64,1.18) | 1.01 (0.78,1.32) |
| Suzhou | 18 | 0.53 (0.34,0.84) | 1.74 (1.23,2.45) | 0.92 (0.62,1.35) | 1.43 (0.88,2.33) |
| Liuzhou | 20 | 0.71 (0.45,1.13) | 0.72 (0.41,1.24) | 1.22 (0.80,1.85) | 1.24 (0.79,1.93) |
| Sichuan | 33 | 1.01 (0.74,1.40) | 1.23 (0.89,1.69) | 1.09 (0.78,1.53) | 1.05 (0.74,1.48) |
| Gansu | 35 | 0.94 (0.65,1.37) | 1.31 (0.94,1.83) | 0.80 (0.58,1.10) | 1.21 (0.80,1.82) |
| Henan | 57 | 1.00 (0.77,1.30) | 1.08 (0.83,1.41) | 0.96 (0.73,1.26) | 0.99 (0.77,1.27) |
| Zhejiang | 18 | 0.63 (0.36,1.10) | 1.07 (0.66,1.74) | 1.06 (0.63,1.77) | 0.60 (0.37,0.98) |
| Hunan | 32 | 0.95 (0.64,1.41) | 1.16 (0.83,1.63) | 0.94 (0.64,1.39) | 1.60 (1.16,2.21) |
| *P* _heterogeneity_ |  | 0.16 | 0.25 | 0.93 | 0.11 |

**Supplementary Fig. 1. The metabolism of n-3 and n-6 PUFAs in human.**

**
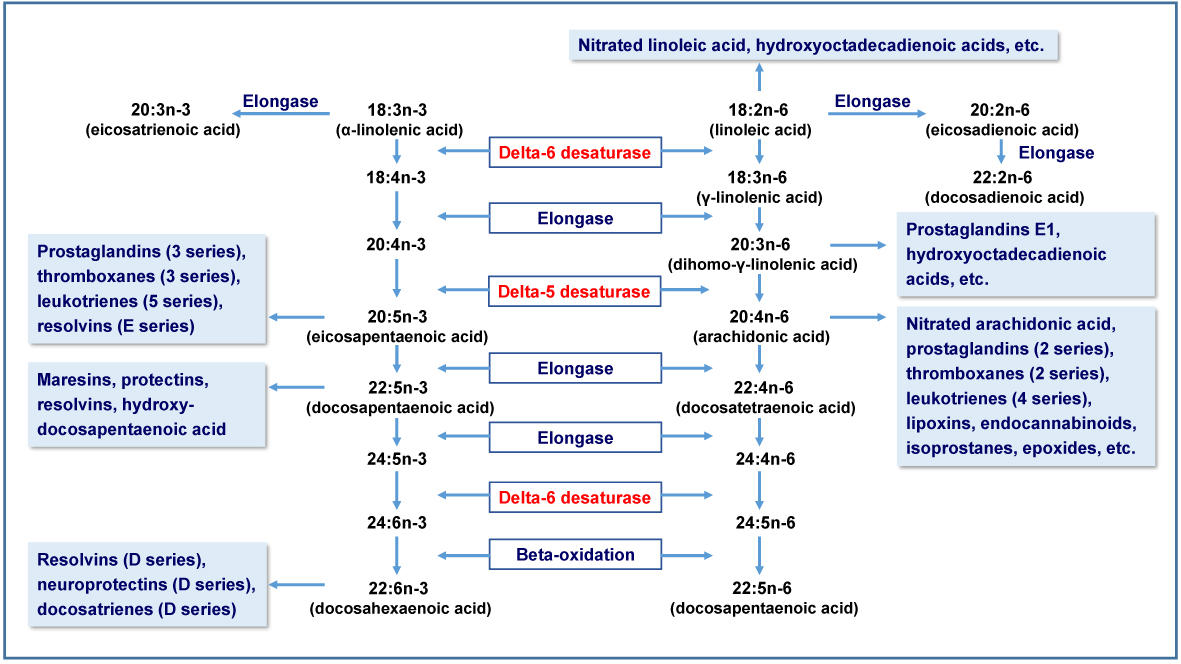
**

**Supplementary Fig. 2. Flow chart of participant selection.**

Participants included in the current analysis (n=8,159)

Participants excluded (n=2,404)

- prevalent vascular diseases or diabetes (n=2,395)
- missing value for blood lipids (n=9)
- No fatty acids measurement (n=14,306)
- Fatty acids data not valid (n=370)

Subsample (n=10,563)

CKB 2013-14 second resurvey examination (n=25,239)

**Supplementary Fig. 3. Median concentrations [Q1;Q3] of major PUFAs by study areas.**


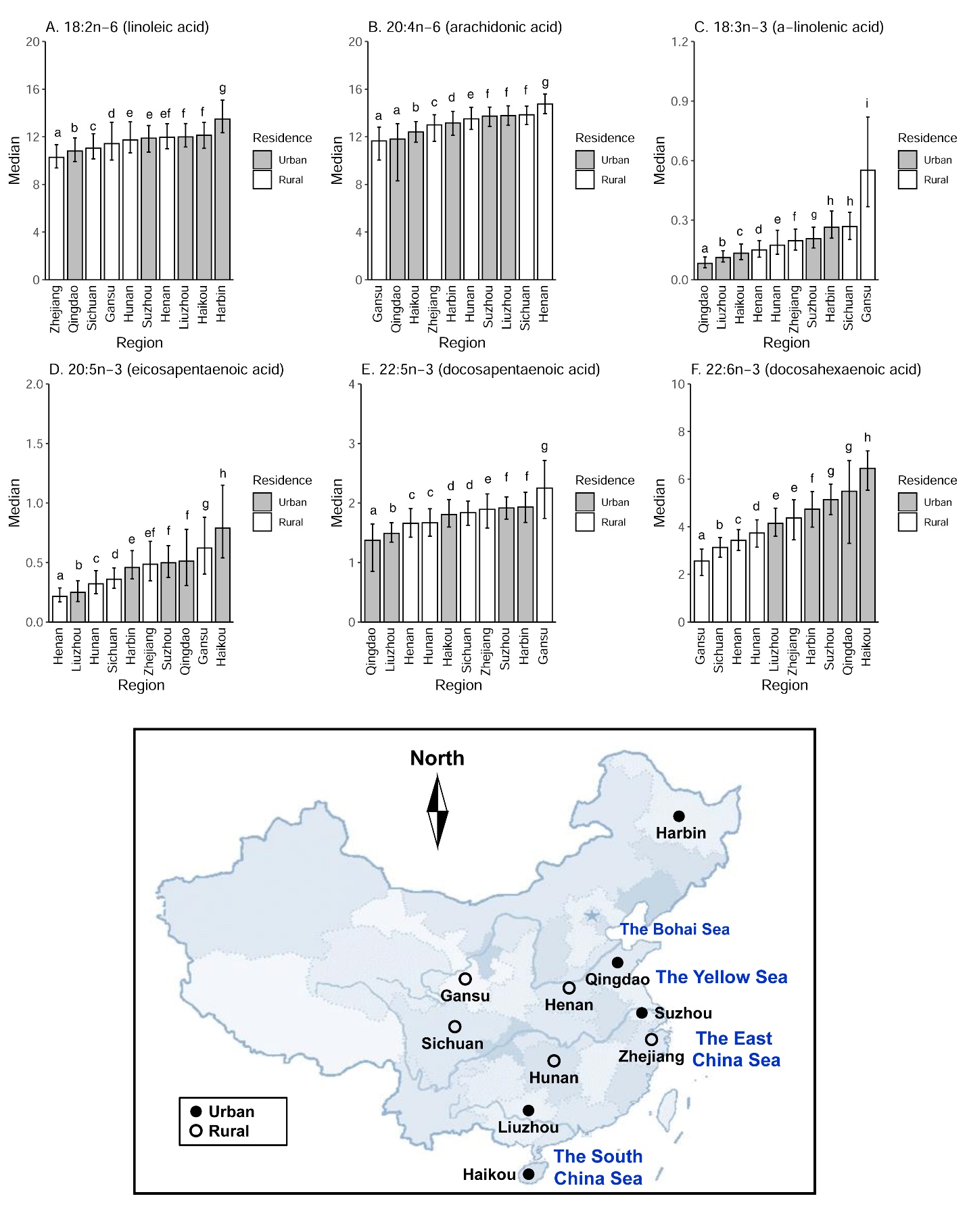


A-F: For each PUFA, columns with same letters are not significantly different from each other.

**Supplementary Fig. 4.** **Associations of erythrocyte PUFAs with risk of total stroke by restricted cubic splines from Cox proportional hazards models.**


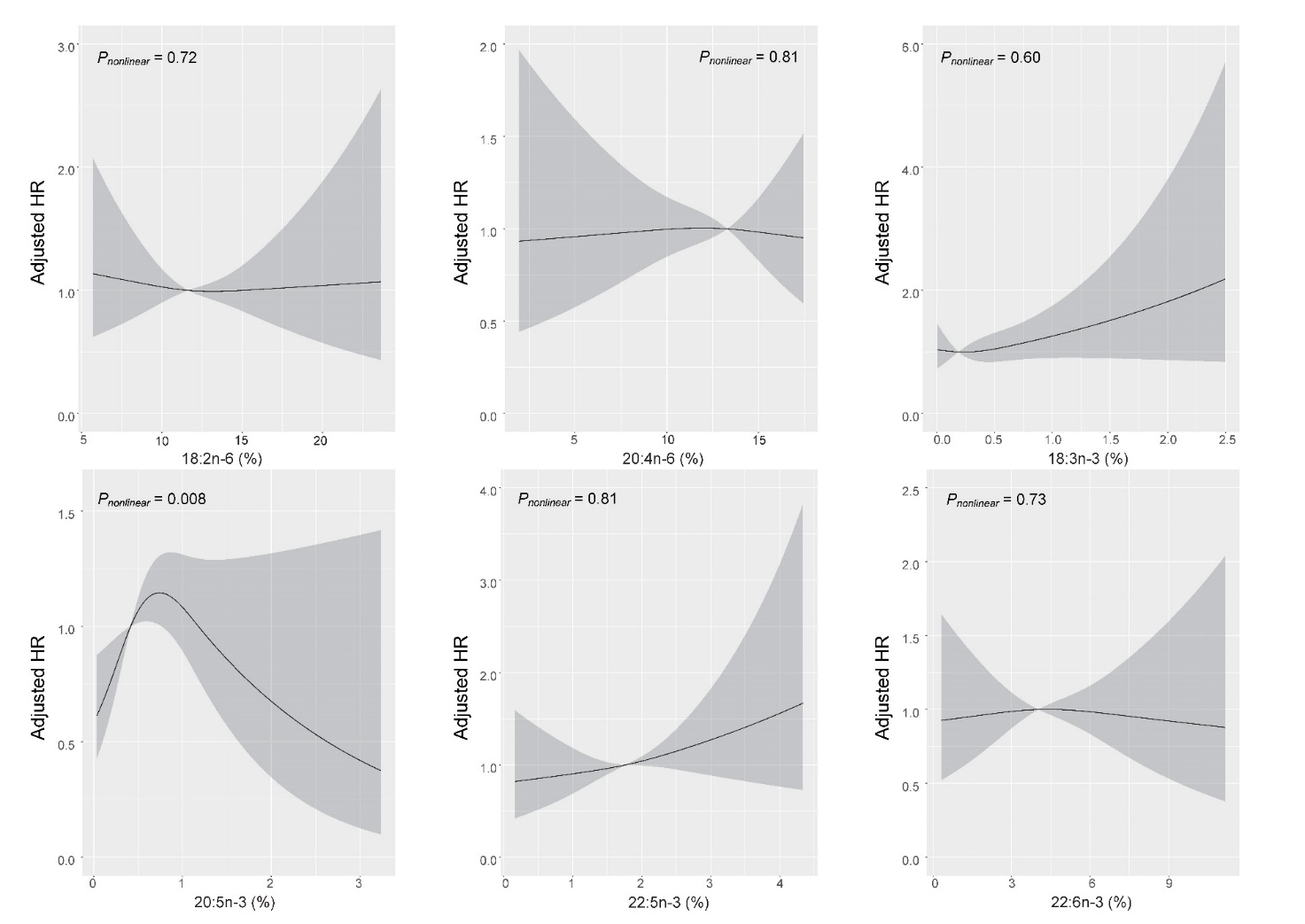


Model was adjusted for age, sex, study areas, education, smoking, alcohol drinking, family history of cardiovascular diseases, and physical activity. The solid lines represent the HRs, and the shaded areas represent 95% Cis, relative to the reference level (50th percentile).
